# miR-455-3p ameliorates pancreatic acinar cell injury by targeting Slc2a1

**DOI:** 10.7717/peerj.15612

**Published:** 2023-06-30

**Authors:** Yinchu Zhan, Chenlin Chen, Zhiqiang Wu, Feng Zhou, Xinping Yu

**Affiliations:** 1 Department of Hepatopancreatobiliary Surgery, The Second People’s Hospital of Quzhou, Quzhou, Zhejiang, China; 2 Department of Clinical Laboratory, The Second Affiliated Hospital of Zhejiang Chinese Medical University, Hangzhou, Zhejiang, China

**Keywords:** Acute pancreatitis, miR-455-3p, Slc2a1, Mouse pancreatic acinar cells, Bioinformatics analysis

## Abstract

**Objective:**

With the number of patients with acute pancreatitis (AP) increasing year by year, it is pressing to explore new key genes and markers for the treatment of AP. miR-455-3p/solute carrier family 2 member 1 (Slc2a1) obtained through bioinformatics analysis may participate in the progression of AP.

**Materials and Methods:**

The C57BL/6 mouse model of AP was constructed for subsequent studies. Through bioinformatics analysis, the differentially expressed genes related to AP were screened and hub genes were identified. A caerulein-induced AP animal model was constructed to detect the pathological changes of mouse pancreas by HE staining. The concentrations of amylase and lipase were measured. Primary mouse pancreatic acinar cells were isolated and subjected to microscopy to observe their morphology. The enzymatic activities of trypsin and amylase were detected. The secretion of inflammatory cytokines in mouse were measured with the ELISA kits of TNF-*α*, IL-6 and IL-1*β* to determine pancreatic acinar cell damage. A binding site between the Slc2a1 3′ UTR region and the miR-455-3p sequence was verified by dual-luciferase reporter assay. The expression of miR-455-3p was quantified by qRT-PCR, and Slc2a1 were detected by western blot.

**Results:**

A total of five (Fyn, Gadd45a, Sdc1, Slc2a1, and Src) were identified by bioinformatics analysis, and miR-455-3p/Slc2a1 were further studied. HE staining results showed that the AP models were successfully established by caerulein induction. In mice with AP, the expression of miR-455-3p was reduced, while that of Slc2a1 was increased. In the caerulein-induced cell model, the expression of Slc2a1 was significantly reduced after intervention of miR-455-3p mimics, whereas increased after miR-455-3p inhibitor treatment. miR-455-3p decreased the secretion of inflammatory cytokines in the cell supernatant, reduced the activity of trypsin and amylase, and alleviated the cell damage induced by caerulein. In addition, Slc2a1 3’UTR region was bound by miR-455-3p, and its protein expression was also regulated.

**Conclusion:**

miR-455-3p alleviated caerulein-induced mouse pancreatic acinar cell damage by regulating the expression of Slc2a1.

## Introduction

Acute pancreatitis (AP), as an inflammatory disease of the digestive tract, tends to show some pathological features, such as spectral edema, vascular accumulation, and inflammatory infiltration of immune cells. Increased secretion of inflammatory cytokines is often accompanied by acute abdominal pain. The incidence of AP has continued to rise in recent years (James & Crockett, 2018; Pandol et al., 2007). Based on the 2012 revised Atlanta classification modified by the Acute Pancreatitis Classification Working Group, AP was categorized into two types (interstitial edematous pancreatitis and necrotizing pancreatitis), with its severity classified as mild, moderately severe or severe (Sun et al., 2019). Mild AP does not present with organ failure, and often resolves within a week. Moderately severe AP causes organ failure that can recover within 48 h, while severe AP causes organ failure for more than 48 h, and a mortality rate of 40% (Leppäniemi et al., 2019). AP is prevalent in America, Japan, Europe and other nations, resulting in a huge socio-economic burden, which along with its after-effects are expected to be even aggravated in 2050 (Munigala et al., 2017).

Multiple factors contribute to the occurrence of AP, but its pathogenesis is still unclear. No consensus has been reached based on the multiple studies that have already been conducted. The common cause of AP is the secondary pancreatic duct obstruction due to alcohol abuse, gallstones, and various drugs (Jiang et al., 2020). Currently, combination therapies are used for the treatment of AP, such as nutritional support, protease inhibitors, and analgesia, but no specific treatment is now available (Zhou et al., 2022). Patients with AP still have poor prognosis due to the circumscribed efficacy of regular therapies and the absence of effectual targets for the treatment of AP. Therefore, it is urgent to find ways to effectively treat or improve AP.

MicroRNA (miRNA) is a protein regulatory factor, which is crucial in the regulation of cellular function and takes part in the modulation of inflammatory responses. AP is an inflammatory disease influenced by miRNA. It has been proposed that miR-155 targets the suppressor of cytokine signaling1 (SOCS1) to regulate Th17/Treg ratio and participates in the development of AP (Wang et al., 2018). The deletion of miR-21 alleviates the lung injury caused by caerulein-induced AP by inhibiting the expression of high mobility group box 1 (Hmgb1), thus protecting mice from AP damage (Li et al., 2018; Ma et al., 2015).

Datasets used in the current study were downloaded from the Gene Expression Omnibus (GEO) database. Bioinformatics analyses including differential gene expression analysis, functional pathways enrichment analysis, and protein-protein interaction (PPI) network analysis were respectively conducted. The regulatory relationship between miRNAs and hub genes was clarified, and miR-455-3p/solute carrier family 2 member 1 (Slc2a1) were finally selected to be investigated in this study. Subsequently, the relationship and role of miR-455-3p and Slc2a1 was verified at the animal and cell levels. This study provides some ideas and certain direction for research on new therapeutic or preventive targets of AP.

## Material and Methods

### Data source

A miRNA-seq dataset (GSE81260) and two mRNA expression datasets (GSE109227 and GSE161945) were obtained from the GEO database (Dixit et al., 2016; Norberg et al., 2018; Wang et al., 2021). GSE81260 contains 15 samples of mice (three AP samples and three control samples were selected). GSE109227 contains 11 samples of mice, including five controls and six AP samples. GSE161945 contains six samples of mice, including three controls and three AP samples.

### Acquisition of differentially expressed genes (DEGs)

The differentially expressed miRNAs (DEmiRs) of GSE81260 and the DEGs of GSE109227 and GSE161945 were analyzed by Limma, and then the DEGs of GSE109227 and GSE161945 were intersected (Ritchie et al., 2015).

### miRNA-mRNA regulatory network and functional pathway enrichment analysis of target genes

Target genes were predicted by miRWalk 3.0 (Huai et al., 2020). The predicted results were intersected with DEGs to acquire the differentially expressed target genes. The pairs with opposite expression trends were selected to establish miRNA-mRNA regulatory network, and were visualized using Cytoscape. Then, Gene Ontology (GO) and Kyoto Encyclopedia of Genes and Genomes (KEGG) analysis of the target genes were conducted using clusterProfiler to obtain functional pathways (Chen et al., 2022).

### PPI network construction

The Search Tool for the Retrieval of Interacting Genes (STRING) database was applied to conduct PPI network analysis of the target genes, and hub genes were picked out by means of Maximal Clique Centrality (MCC), Edge Percolated Component (EPC) and Degree through cytohubba plug-in (Xie et al., 2022). Then, the regulatory relationship between miRNAs and hub genes was further analyzed, and their expression was also detected.

### Construction of mouse AP model

The Laboratory Animal Center of Hangzhou Medical College provided SPF grade C57BL/6 male mice for experiments, and the experimental protocols were permitted by the Institutional Animal Care and Use Committee (IACUC), Zhejiang Center of Laboratory Animals (ZJCLA) (Approval No. ZJCLA-IACUC-20040130). Healthy 8-week-old C57BL/6 mice (20–22 g) were kept in an air-conditioned room with suitable room temperature and humidness. Before animal modeling, the mice were adapted to the facility for 1 week, and fed with commercial rodent pellets.

The C57BL/6 mice were equally and randomly grouped into control and AP groups, with six mice in each group. Before modeling, mice in both groups were fasted for more than 8 h, during which their access to drinking water was not restricted. The treatment methods of mice were as follows: Mice in the AP group were injected intraperitoneally with caerulein, repeated 6 times, 1 h each time, and the dose was 100 µg/kg. Mice in the control group were injected with normal saline, repeated 6 times at an interval of 1 h (Zhu, Liu & Wang, 2020).

### Pathological analysis of AP mice

The experimental mice were euthanized through administration of pentobarbital and cervical dislocation, and the serum was collected for subsequent testing. The Olympus Au2700 system, the MODULAR P800 automatic biochemical analyzer, and their companion reagents were applied to measure the contents of amylase and lipase. After staining the pancreatic tissues with HE, two pathologists applied a microscope to evaluate the pathological changes in a blinded manner. The pancreatic edema, immune cell infiltration and acinar necrosis were also graded (0-3), and the pathological grading standards are shown in Table 1. Mice with each pathological grade >1 were used for analysis (Miao et al., 2019).

**Table 1:** Pathological scoring criteria.

**Condition**	**Score**	**Description**
Edema	0	Absent
	1	Diffuse expansion of interlobular septa
	2	1+ diffuse expansion of interlobular septa
	3	≥2+ diffuse expansion of interlobular septa
Inflammation (%)	0	Absent
	1	Around ductal margin
	2	In parenchyma (<50 of lobules)
	3	In parenchyma (≥51 of lobules)
Vacuolization (%)	0	Absent
	1	Periductal (<5)
	2	Focal and Diffuse (5–50)
	3	Severe (>50)

### Isolation of pancreatic acinar cells

Mice were euthanatized, and the abdomen was disinfected with 75% alcohol. Laparotomy was performed to remove the intestinal tract and expose the pancreas and spleen. Then, the pancreas was taken out, and the blood vessels and adipose tissues were removed in precooled DMEM/F12 medium. The pancreas was cut open, and subsequently, the tissue fragments underwent a 30-minute digestion period with the collagenase digestion solution at 37 °C. During this process, the mixture was intermittently agitated every 5 min to ensure thorough digestion. Following the completion of digestion, the resulting digestive juice was transferred into a centrifuge tube, centrifuged at 1,000 rpm for 3 min, and the supernatant was abandoned. The pancreatic acinar cells were resuspended with HEPES buffer, subjected to gentle agitation and then precipitated. After standing for 30s, the buffer was filtered by 150 mesh filter and transferred to 45 mL centrifuge tube. This process was repeated 3 times. Cells were centrifuged at 500 rpm for 3 min, and the supernatant was discarded. Finally, the pancreatic acinar cells were resuspended with DMEM/F12 supplemented with 10% fetal bovine serum (FBS) in preparation for subsequent experiments (Norberg et al., 2018).

### Transfection assay of pancreatic acinar cells

Pancreatic acinar cells were transfected with miRNA-NC, miR-455-3p mimics, miR-455-3p inhibitor, siNC and siSlc2a1 (Shanghai GenePharma Co., Ltd., Shanghai, China) using 0.25% Lipofectamine 2000 (Invitrogen, Waltham, MA, USA). Their transfection concentration is 60nM, and the transfection time is 24 h (Zhang et al., 2019). The sequences of the oligonucleotides were showed as follows: miR-455-3p mimics: GCAGUCCACGGGCAUAUACAC; miR-455-3p inhibitor: CGUCAGGUGCCCGUAUAUGUG.

### Detection of cytokines

The contents of TNF-*α*, IL-1 *β*, and IL-6 in the serum and pancreatic acinar cell supernatant were analyzed using the ELISA kits (R & D Systems, Minneapolis, MN, USA) as per the instructions. Then, the protein secretion was calculated based on the standard curve.

### Detection of intracellular trypsin

The pancreatic acinar cells were grouped and treated as follows: Control: no treatment. Caerulein: pancreatic acinar cells were induced with caerulein (1 × 10^−7^M) for 5 h (Zhang et al., 2022). Caerulein + miRNA-NC group: pancreatic acinar cells were first treated with miRNA-NC transfection, and then stimulated with caerulein. Caerulein + miR-455-3p mimics group: pancreatic acinar cells were first treated with miR-455-3p mimics transfection, and then stimulated with caerulein. After the cells were digested by trypsin, the liquid was centrifuged, and the cells precipitates were collected. Then, the pancreatic acinar cells were resuspended with HEPES buffer containing 2mM EDTA (HBS, 5mM HEPES, 0.15M NaCl, PH 7.35). The cell density was controlled at 1 × 10^6^ cells/mL (Zhang et al., 2019), and 10 µM Rhodamine 110, bis-(p-tosyl-L-glycyl-L-prolyl-L-arginine amide) (R22124; Molecular Probes, Eugene, OR, USA) was added for 20 min of incubation. The fluorescence intensity was detected by laser confocal microscopy and the activity of trypsin was analyzed.

### Western blot assay

The RIPA lysis buffer was used to extract proteins from pancreatic acinar cells. The proteins underwent SDS-PAGE, and were transferred onto polyvinylidene fluoride (PVDF) membranes. Then, the membranes were sealed in skim milk for 2 h. The primary antibodies (1:1000) was applied to incubate proteins overnight. Next day, the secondary antibody was used to incubate the membrane for 2 h. The ECL kit was used to evaluate the chemical luminescence intensity. The primary antibodies used were Slc2a1 (73015S; Cell Signaling Technology (CST), Danvers, MA, USA) and *β*-actin (4970S; CST, Danvers, MA, USA).

### qRT-PCR

Total RNA of the samples was obtained using the RNA extraction kit (R1200; Solarbio, Beijing, China). The cDNA used for qPCR was obtained by reverse transcription. Subsequently, the qPCR experiments were performed by the use of SYBR Premix Ex Taq II (RR820A; TaKaRa, Shiga, Japan) on a real-time PCR system. The primer sequences are showed as follows: miR-455-3p forward primer (F-primer): CGGCAGTCCACGGGCAT; reverse primer (R-primer): AGTGCAGGGTCCGAGGTATT; the internal control gene U6 F-primer: CTCGCTTCGGCAGCACA; R-primer: AACGCTTCACGAATTTGCGT. The miR-455-3p expression was calculated by 2^−ΔΔ*Ct*^ method.

### Assay of amylase activity

The amylase activity in the supernatant was detected based on the instructions of EnzChek Ultra Amylase Assay Kit (E33651; Molecular Probes, Eugene, OR, USA). The relative fluorescence intensity of each well was detected by a microplate detector. The maximum absorption wavelength was set at 505nm and the maximum emission wavelength was set at 512 nm.

### Dual-luciferase reporter assay

The pGL3-Slc2a1-3′-UTR (WT) or pGL3-Slc2a1 mut- 3′-UTR (MT) and miRNA-NC or miR-455-3p mimics were transfected into 293T cells, respectively. Following the instructions, the dual-luciferase reporter gene detection kit (FR201-01; Beijing Trans Gen Biotech, Beijing, China) was applied to calculate the relative luciferase activity. The sequences are as follows: Slc2a1-3′ UTR: TGTGTGTGTGCTTACAGAGT; Slc2a1-mut-3′ UTR: TCACATATACGTTACAGAGT.

### Statistical analysis

GraphPad Prism 8 (GraphPad Inc., San Diego, CA, USA) was used to analyze the experimental data. Comparisons between groups were assessed by Student’s *t* test, and one-way ANOVA, followed by Bonferroni test for the difference comparison among multiple groups. Data was showed as mean ± SD. At least three biological replicates were performed for each assay. *P* < 0.05 was judged to be statistically significant.

## Results

### Acquisition of DEGs

**Figure 1:**
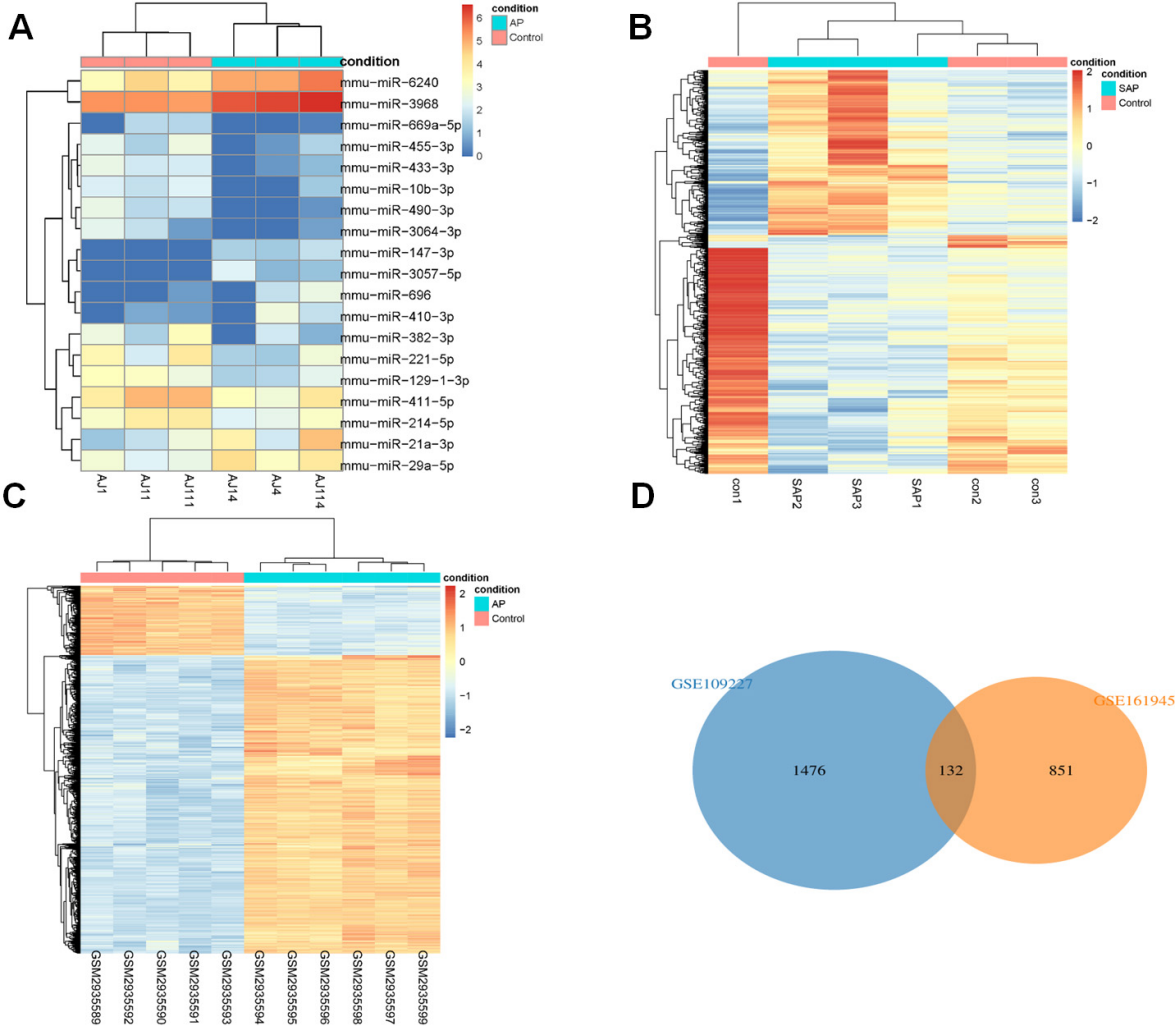
DEGs analysis. (A) Heat-map of DEmiRs in GSE81260; (B) Heat-map of DEGs in GSE161945; (C) Heat-map of DEGs in GSE109227; (D) Venny diagram of DEGs in GSE161945 and GSE109227.

DEGs analysis of Limma (|logFC| > 1, *P* < 0.05) identified 19 DEmiRs in GSE81260, and there were 8 up-regulated miRNAs and 11 down-regulated miRNAs (Fig. 1A). 983 DEGs were acquired in GSE161945, of which 402 DEGs were up-regulated and 581 DEGs were down-regulated (Fig. 1B). Among 1608 DEGs found in GSE109227, 1304 were up-regulated and 304 were down-regulated (Fig. 1C). Through Wayne diagram analysis, 132 DEGs were found in GSE161945 and GSE109227 datasets (Fig. 1D).

### Functional enrichment analysis (GO and KEGG)

**Figure 2:**
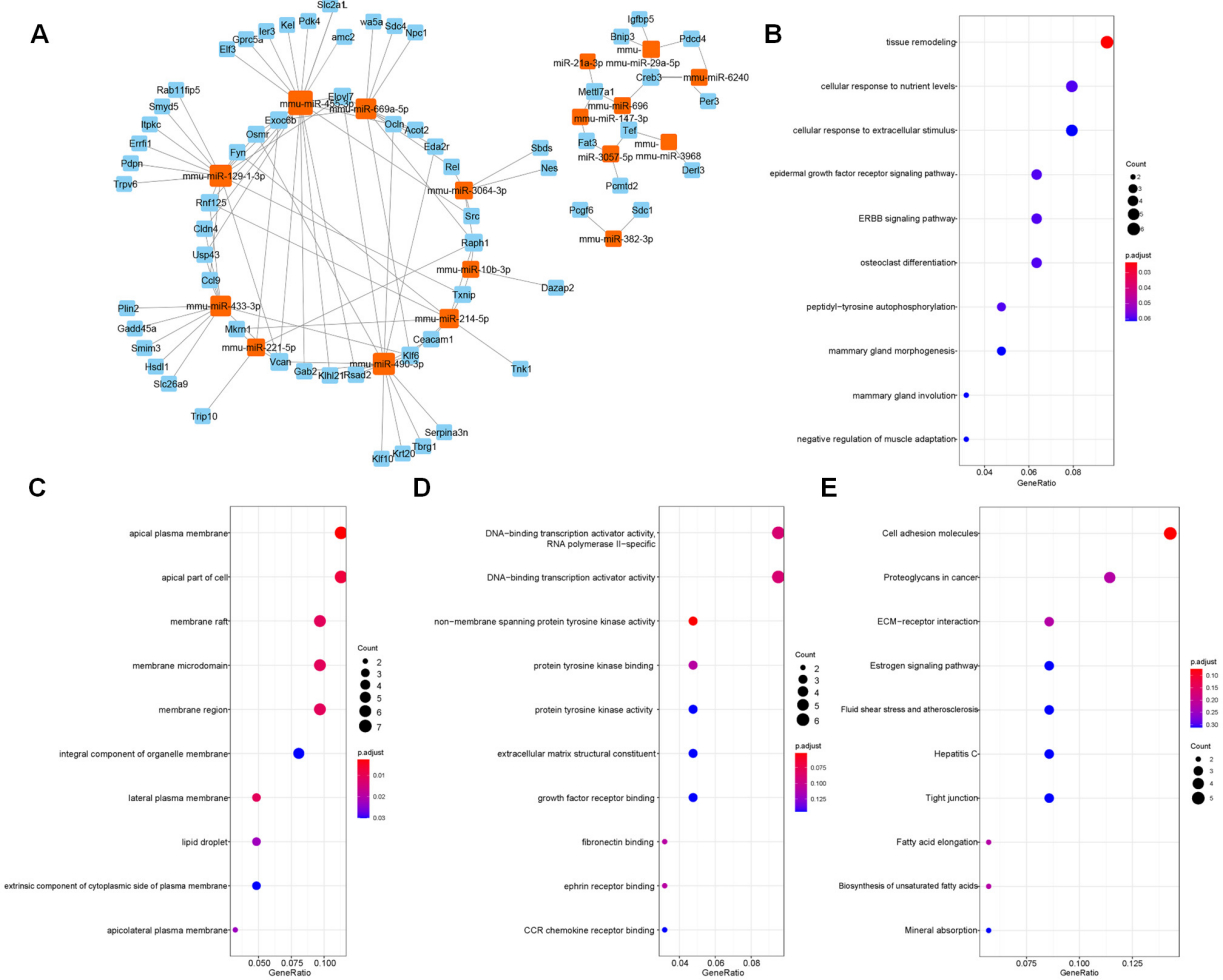
Screened target genes and functional enrichment analysis. (A) The regulatory network of miRNA-mRNA; (B–E) The functional pathways enrichment analysis (GO-BP, GO-CC, GO-MF and KEGG); BP, biological process; CC, cell component; MF, molecular function.

The target genes predicted by miRWalk 3.0 were crossed with 132 DEGs, and 88 DEGs were preserved. Only the opposite relationship between miRNA and mRNA expression was retained. Finally, 110 targeted relationships between 17 miRNAs and 64 mRNAs were obtained (Fig. 2A). GO and KEGG were then used to analyze the functional enrichment of the target genes. It was found that the functions of the target genes were primarily enriched in tissue remodeling, cytoplasmic membrane, nutrient interaction of cells, and non-membrane transmembrane tyrosine kinase activity (Figs. 2B–2E).

### PPI network analysis

**Figure 3:**
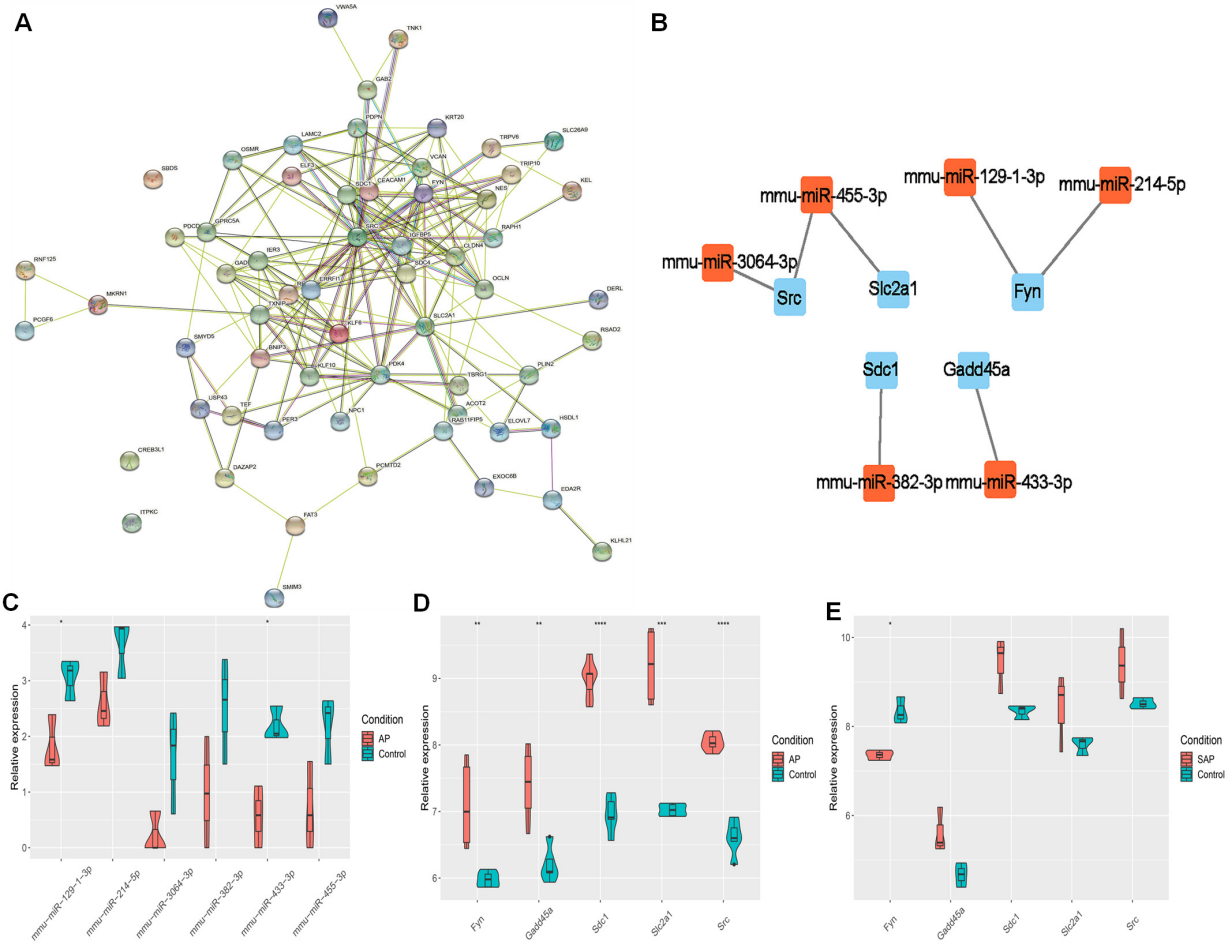
The regulatory relationship between hub genes and miRNAs. (A) A PPI network; (B) Regulatory relationship between miRNA and hub genes; (C) miRNA expression in AP and Control groups; (D–E) Expression of five hub genes in GSE109227 and GSE161945.

The target genes were subjected to PPI network analysis by STRING, and a network with 58 nodes and 197 edges was obtained (Fig. 3A). 5 hub genes (Fyn, Gadd45a, Sdc1, Slc2a1 and Src) were identified by MCC, EPC and degree algorithms (Table 2). The regulatory relationship between miRNAs and 5 hub genes (Fig. 3B) were revealed. Meanwhile, their expression was analyzed. miR-3064-3p, miR-455-3p, miR-129-1-3p, miR-214-5p, miR-382-3p, and miR-433-3p were down-regulated in AP (Fig. 3C). In GSE109227 and GSE161945, except Fyn, the expressions of Gadd45a, Sdc1, Slc2a1 and Src were up-regulated in AP (Figs. 3D–3E). In this study, miR-455-3p/Slc2a1 was further studied.

**Table 2:** Hub genes.

**Methods**	**MCC**	**EPC**	**Degree**
Top 10 hub genes	Src	Src	Src
	Sdc1	Igfbp5	Slc2a1
	Nes	Slc2a1	Pdk4
	Slc2a1	Fyn	Igfbp5
	Ocln	Sdc1	Fyn
	Fyn	Nes	Errfi1
	Vcan	Pdk4	Cldn4
	Gadd45a	Errfi1	Sdc1
	Ier3	Ocln	Rel
	Sdc4	Gadd45a	Gadd45a

### Successful construction of mouse AP model

**Figure 4:**
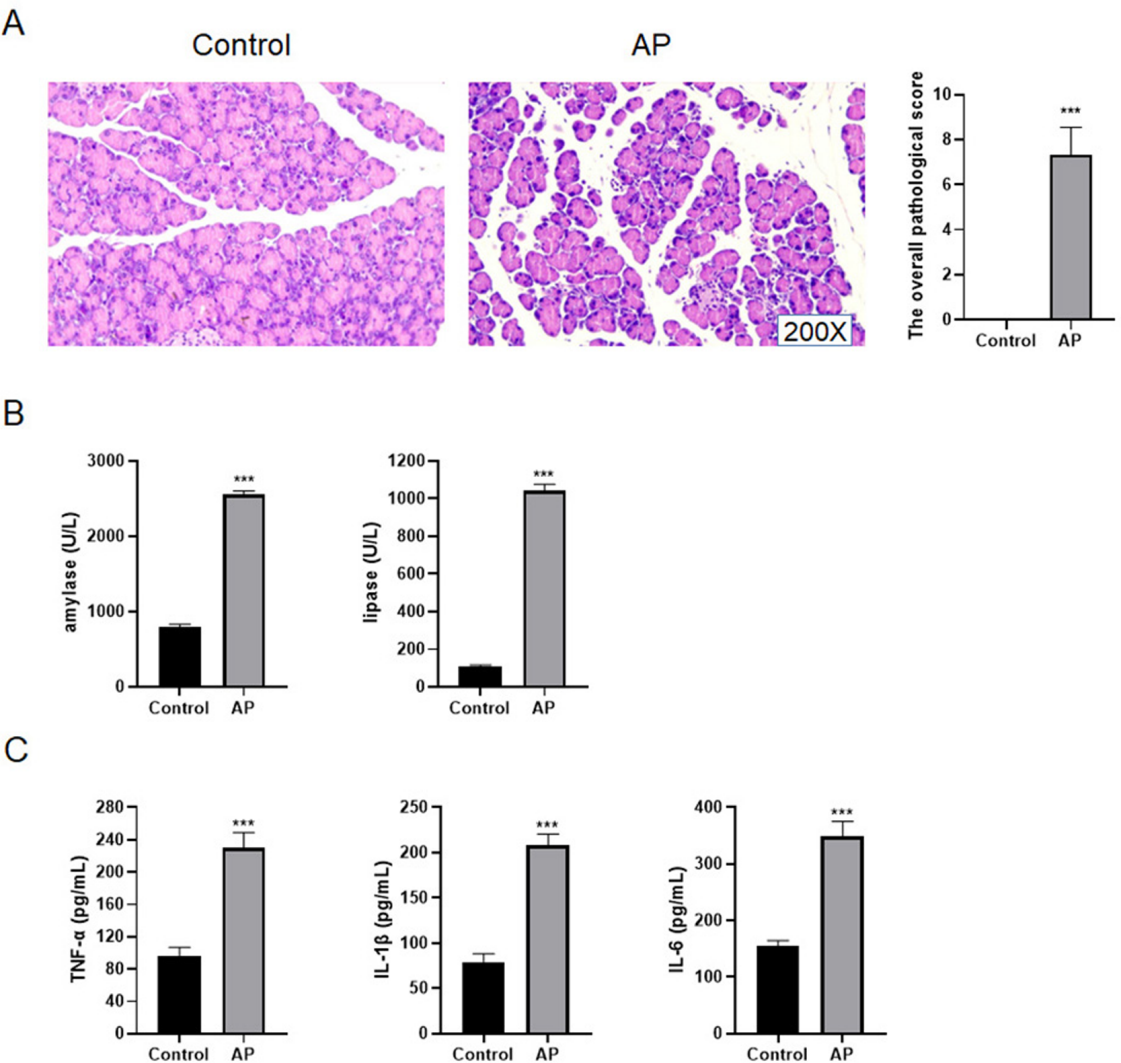
Construction of mouse AP model. (A) HE staining and the overall pathological score; (B) Amylase and lipase in mice serum; (C) Detection of serum inflammatory cytokines by ELISA (^***^P < 0.001).

In order to identify whether the mouse AP model was successfully built, the pancreas tissues of C57BL/6 mice were taken for HE staining. The results presented that the pancreatic lobules of control mice were clear, without edema, bleeding, inflammatory cell infiltration and necrosis, and its overall pathological score was 0 (Fig. 4A). For AP mice, the pancreatic lobule space widened, the acinar edema, flake necrosis, and a great quantity of immune cell infiltration were noticed in the parenchyma and stroma, indicating that red blood cells were scattered in the pancreatic tissues. The overall pathological score was 6.81 ± 0.67 (Fig. 4A). Furthermore, the serum amylase and lipase contents of AP mice increased appreciably as compared with control mice (Fig. 4B). The contents of TNF-*α*, IL-1 *β* and IL-6 in AP mice were significantly higher than those of control mice in the serum (Fig. 4C).

### miR-455-3p and Slc2a1 in AP mice

**Figure 5:**
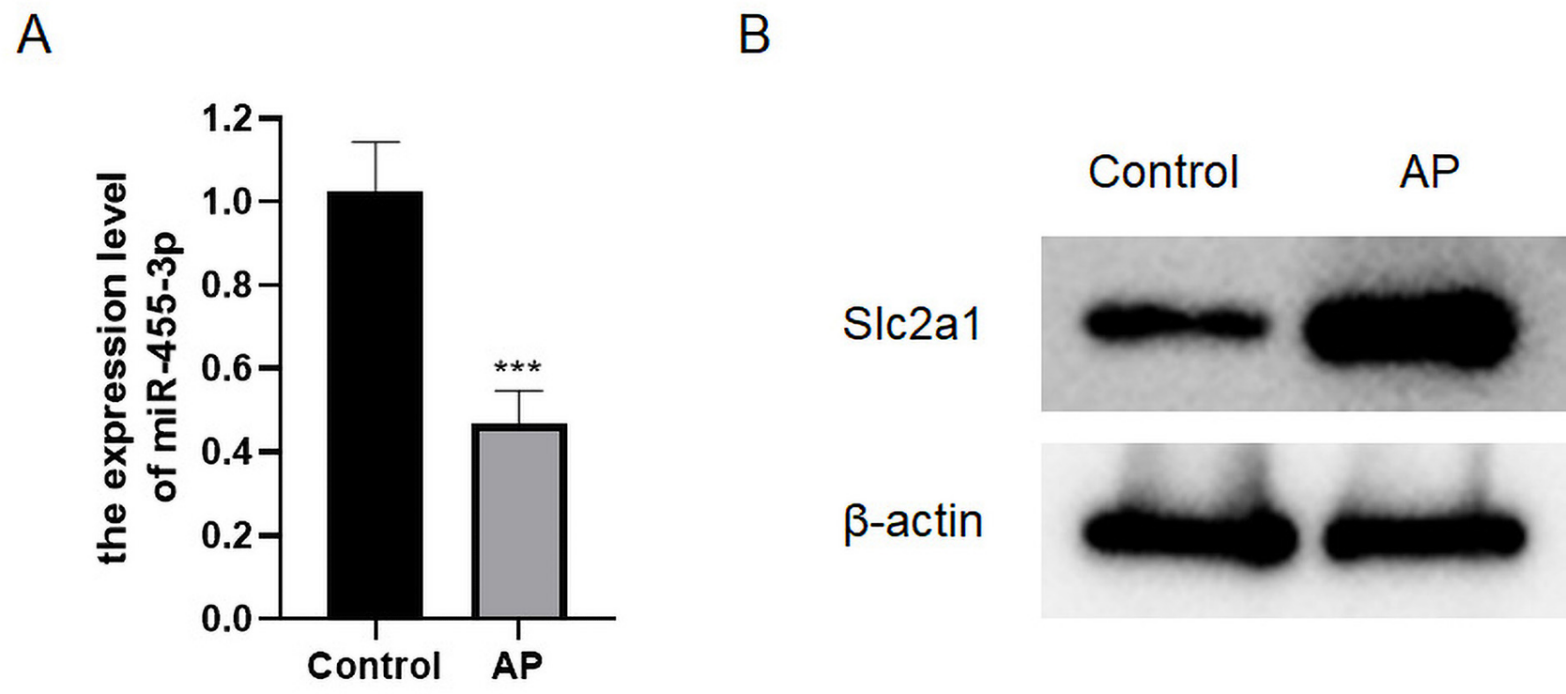
Expression of miR-455-3p and Slc2a1. (A) Detection of miR-455-3p by qRT-PCR; (B) Detection of Slc2a1 by western blot (^***^P < 0.001).

qRT-PCR was applied to detect the level of miR-455-3p, and western blot was used to detect Slc2a1 in the mouse pancreatic tissues (Figs. 5A–5B). In contrast to control mice, miR-455-3p of AP mice was significantly down-regulated in the pancreatic tissues, while Slc2a1 protein was significantly increased, which was consistent with the findings of bioinformatics analysis.

### miR-455-3p attenuates caerulein-induced pancreatic acinar cell injury

**Figure 6:**
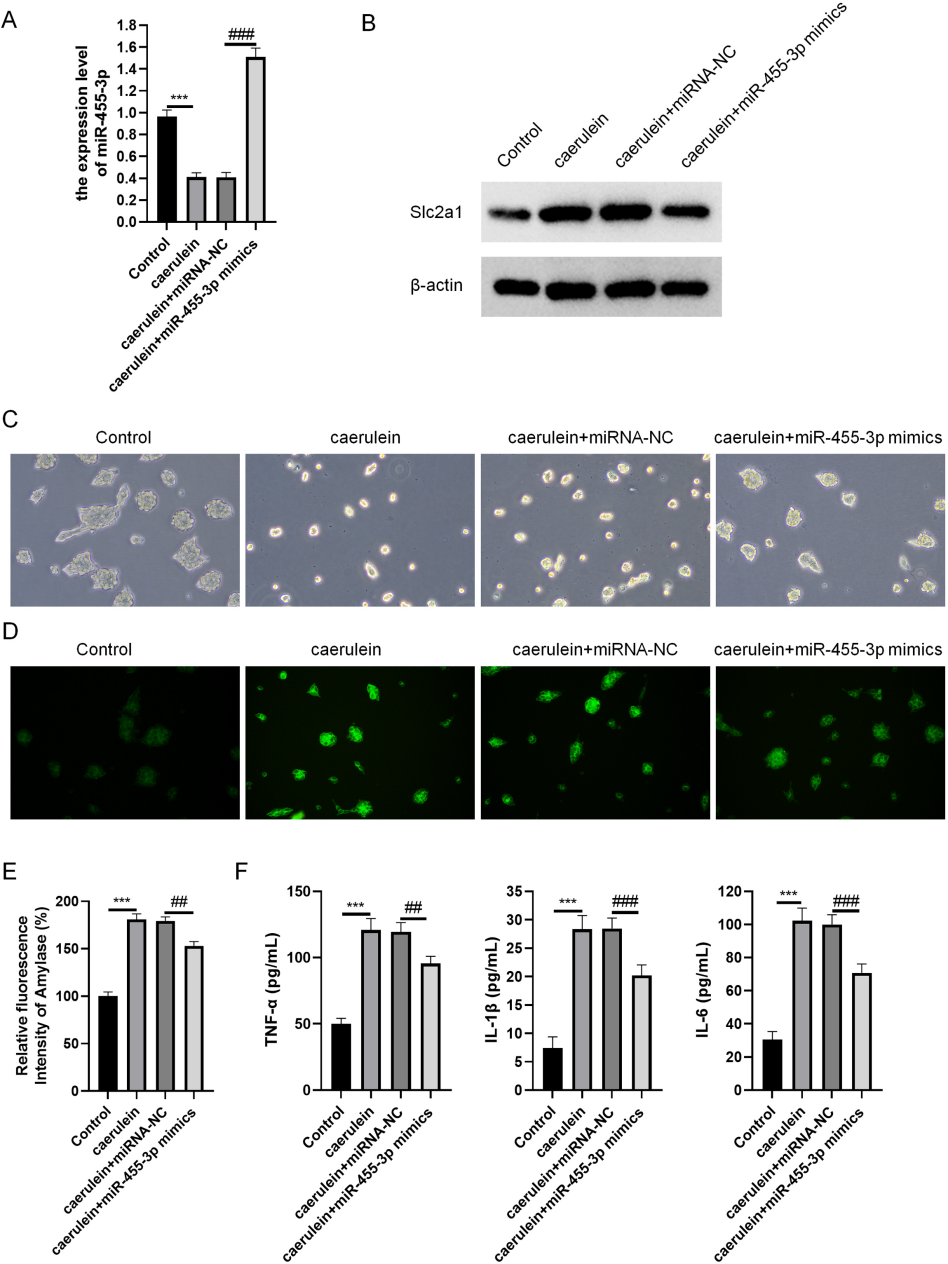
miR-455-3p alleviated the caerulein-induced pancreatic acinar cell injury. (A) Measurement of miR-455-3p; (B) Detection of Slc2a1 by western blot; (C) Morphological observation of pancreatic acinar cells under the optical microscope; (D) Detection of trypsin activity by Rhodamine 110 staining kit; (E) Amylase in the cell supernatant; (F) Detection of inflammatory cytokines (Compared with the control, ^***^P < 0.001; compared with the caerulein + miRNA-NC, ^##^*P* < 0.01, ^###^*P* < 0.001).

miR-455-3p and Slc2a1 were detected through qRT-PCR and western blot (Figs. 6A–6B). The cell morphology of each group was observed under an optical microscope (Fig. 6C). When compared with cells in the control, the density of caerulein-induced cells was obviously down-regulated, the number of bright and transparent normal cells was reduced, the cells were seriously damaged, the shape was irregular, and there were more suspended dead cells and cell debris. Compared with the caerulein + miRNA-NC, the cell damage was improved in the caerulein + miR-455-3p mimics, the cell shape was relatively regular, and the proportion of dead cells and cell debris were significantly reduced. The cells were stained with Rhodamine 110 staining kit, and a laser confocal microscope was applied for observation (Fig. 6D). The positive region of trypsin (green fluorescence) in the caerulein-induced cells was obviously increased compared with the control. Compared to the caerulein + miRNA-NC, the trypsin activity in caerulein + miR-455-3p mimics group decreased. Amylase is another common marker of pancreatic diseases. Therefore, the amylase activity detection kit was applied to analyze the amylase in the cell culture supernatant (Fig. 6E). Compared to the control, the amylase activity in the cell culture supernatant of the caerulein group was increased (Fig. 6E). The amylase activity in the caerulein + miR-455-3p mimics was down-regulated than the caerulein + miRNA-NC group (Fig. 6E). The secretion level of inflammatory cytokines (IL-1 *β*, IL-6 and TNF-*α*) was analyzed by ELISA (Fig. 6F). The secretion level of inflammatory cytokines in the cell culture supernatant of caerulein-induced cells were obviously increased than the control, while substantially reduced in the caerulein + miR-455-3p mimics by contrast to the caerulein + miRNA-NC group.

### miR-455-3p directly regulates Slc2a1

**Figure 7:**
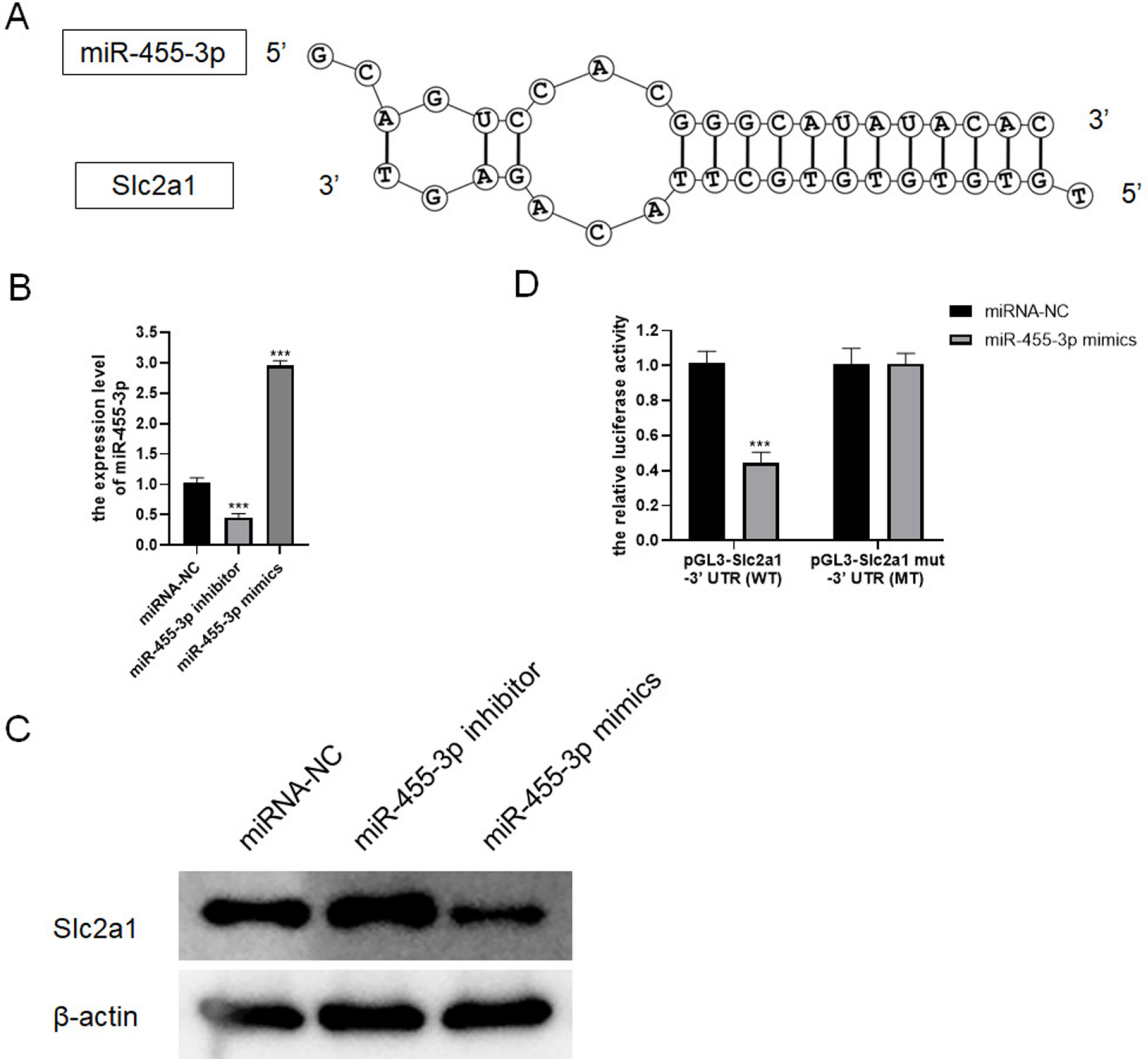
miR-455-3p targeted Slc2a1. (A) The binding site of miR-455-3p to Slc2a1 3′ UTR region; (B) Measurement of miR-455-3p; (C) Detection of Slc2a1 by western blot; (D) The experimental results of dual-luciferase reporter assay (^***^P < 0.001).

Based on bioinformatics analysis, the binding sites to the miR-455-3p sequence exist in the Slc2a1 3′ UTR region (Fig. 7A). In primary mouse pancreatic acinar cells, Slc2a1 expression was significantly reduced by miR-455-3p mimics but markedly enhanced by miR-455-3p inhibitor (Figs. 7B–7C). In addition, the luciferase reporter vector was applied to further identify the combination between miR-455-3p and Slc2a1 3′ UTR region. After co-transfection of miR-455-3p mimics and pGL3-Slc2a1-3′-UTR (WT) plasmids in 293T cells, it was found that the luciferase activity was significantly decreased (Fig. 7D). There is no significant difference between pGL3-Slc2a1 mut-3′-UTR (MT)/miRNA-NC and pGL3-Slc2a1 mut-3′-UTR (MT)/miR-455-3p mimics (Fig. 7D).

### miR-455-3p targets Slc2a1 and alleviates the damage of mouse pancreatic acinar cells induced by caerulein

**Figure 8:**
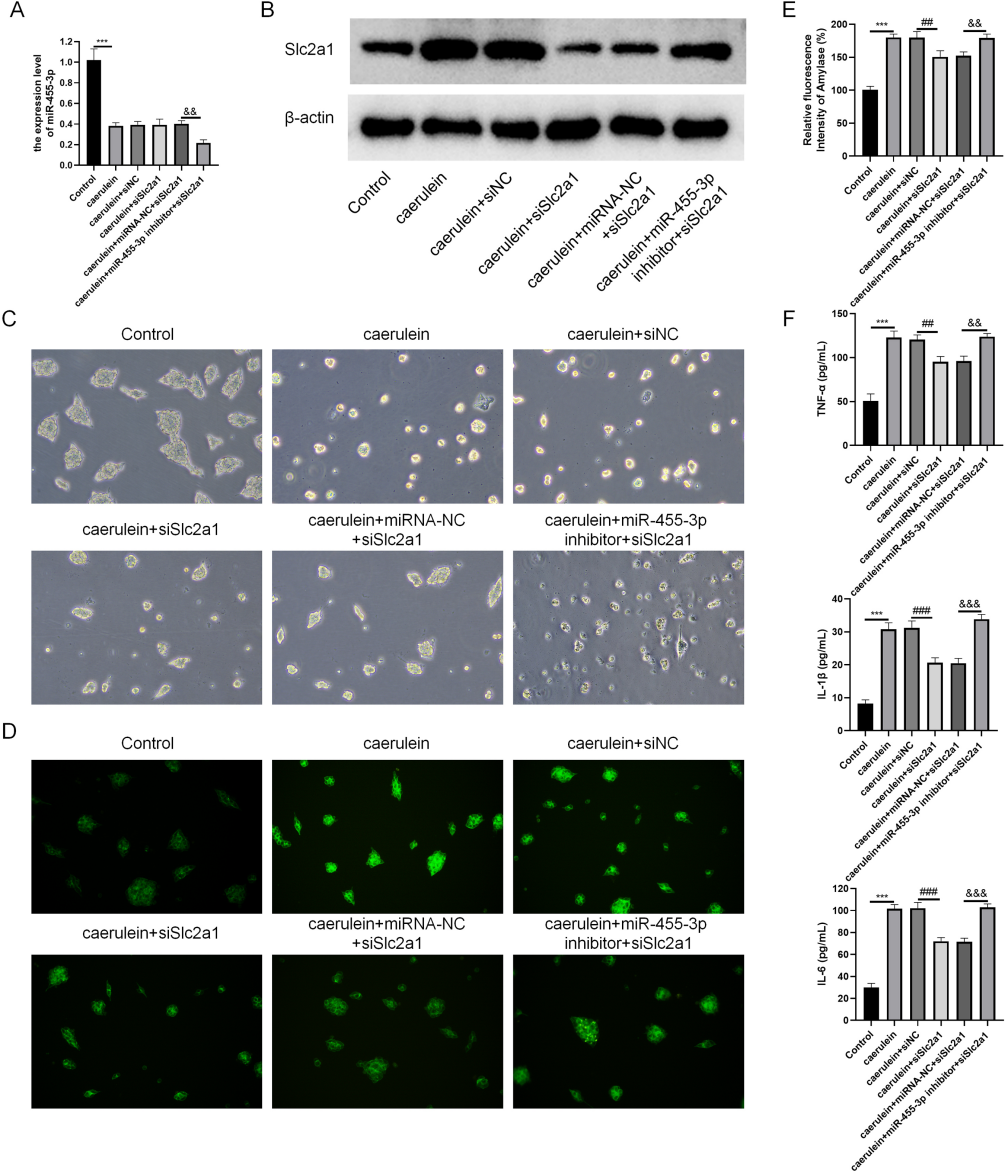
miR-455-3p targeted Slc2a1 to alleviate the caerulein-induced pancreatic acinar cell injury. (A) Detection of miR-455-3p; (B) Detection of Slc2a1 by western blot; (C) Morphological observation of pancreatic acinar cells under the optical microscope; (D) Detection of trypsin activity by Rhodamine 110 staining kit; (E) Amylase in the cell supernatant; (F) Detection of inflammatory cytokines (Compared with the control, ^***^P < 0.001; compared with the caerulein + siNC, ^##^*P* < 0.01, ^###^*P* < 0.001; compared with the caerulein + miRNA-NC + siSlc2a1, ^&&^*P* < 0.01, ^&&&^*P* < 0.001).

Combined with the results of bioinformatics analysis and cell experiments, it was found that miR-455-3p bound to the 3′ UTR region in Slc2a1, thereby regulating the transcript level of Slc2a1 (Figs. 8A–8B). For further verification, siNC, siSlc2a1, miRNA-NC and miR-455-3p inhibitor were used to transfect into primary mouse pancreatic acinar cells. To test whether miR-455-3p alleviates the damage of caerulein-induced mouse pancreatic acinar cells by regulating Slc2a1, cells were grouped into control group, caerulein group, caerulein + siNC group, caerulein + siSlc2a1 group, caerulein + miRNA-NC + siSlc2a1 group, and caerulein + miR-455-3p inhibitor + siSlc2a1 group. It was shown that the caerulein-induced damage of pancreatic acinar cells was improved in the caerulein + siSlc2a1 compared to the caerulein + siNC, and the trypsin and amylase activity and the secretion levels of inflammatory cytokines were decreased (Figs. 8C–8F). Compared to the caerulein + miRNA-NC + siSlc2a1 group, the cell damage in the caerulein + miR-455-3p inhibitor + siSlc2a1 was aggravated, and the trypsin and amylase activity and the secretion levels of inflammatory cytokines were increased (Figs. 8C–8F).

## Discussion

In summary, five hub genes (Fyn, Gadd45a, Sdc1, Slc2a1, and Src) were identified in this study through bioinformatics analysis, and miR-455-3p/Slc2a1 (GLUT1) was further analyzed. Based on *in vitro* and *in vivo* studies, it was confirmed that miR-455-3p targeted Slc2a1, reduced the secretion levels of inflammatory cytokines in the supernatant of caerulein-induced cells, inhibited the trypsin and amylase activity, and alleviated caerulein-induced cell damage. Consequently, miR-455-3p/Slc2a1 could be a therapeutic target of AP.

With an increasing incidence, AP has become one of the major gastrointestinal diseases leading to hospitalization in many countries. At present, on account of the unclear etiology of AP and a lack of effective treatment for AP (Pan et al., 2018), it is of high necessity to explore novel therapeutic targets for this disease. In the current study, datasets were firstly obtained from the online database (GEO), including miRNA-SEQ dataset GSE81260, and mRNA expression dataset GSE109227 and GSE161945. After differential expression analysis of these datasets, target genes were predicted, and 88 differentially expressed target genes were obtained after intersection with DEGs. Functional enrichment analyses (GO and KEGG) of these target genes demonstrated that the functional pathways were mainly centered at tissue remodeling, cytoplasmic membrane, cell nutrient interaction, and non-membrane spanning tyrosine kinase activity. AP often leads to local inflammatory responses of the pancreas at the initial stage, and may eventually lead to systemic inflammation and complications, resulting in tissue damage in individuals (Lankisch, Apte & Banks, 2015). The functions of these target genes may alleviate the damage of cells and tissues in the process of AP, and regulate its severity categories. Finally, we found 5 hub genes (Fyn, Gadd45a, Sdc1, Slc2a1, and Src) and analyzed the regulatory relationship between miRNA and the 5 hub genes. In this research, miR-455-3p/Slc2a1 was further studied.

Previous studies claimed that miR-455-3p is of great importance in esophageal cancer, colorectal cancer, lung cancer, melanoma, and breast cancer (Liu et al., 2017; Ni et al., 2020). Besides, miR-455-3p suppressed pancreatic cancer progression by regulating transcriptional coactivator with PDZ binding motif (Zhan et al., 2020). Nevertheless, the function of miR-455-3p in AP has not been studied yet. miR-455-3p was revealed in the current study to alleviate caerulein-induced damage of the mouse pancreatic acinar cells, and reduce the trypsin and amylase activity. It was also supposed that miR-455-3p might alleviate pancreatic damage in AP. miRNA is a small non-coding RNA containing 22-24 nucleotides, and miRNA targets mRNA 3′ UTR region complementary binding leading to degrade mRNA or inhibit protein translation (Ruksha, Komina & Palkina, 2017). Bioinformatics analysis and dual-luciferase reporter assay demonstrated that Slc2a1 3′ UTR region combined with miR-455-3p sequence.

Slc2a1, which encodes the protein of a crucial glucose transporter (GLUT1), is important in the energy metabolism pathway of cells. It was highly expressed in endometrial cancer, lung cancer, breast cancer, and glioma (Benzerdjeb, Berna & Sevestre, 2017; Shi et al., 2019; Zhao et al., 2019), and could act as a potential biomarker or target for the early diagnosis and precise target treatment of pancreatic ductal adenocarcinoma (Zhou et al., 2019). In the current study, we explored the function of Slc2a1 in AP. Here, miR-455-3p was found to regulate Slc2a1 protein, and Slc2a1 was shown to affect the mouse pancreatic acinar cell damage induced by caerulein. We speculated that Slc2a1 affects glucose transport, as well as glycolysis, which is able to supply the required energy for the secretion of enzymes and cytokines in pancreatic acinar cells.

TNF-*α* and IL-1 *β*, as the main cytokines in the progression of AP, can trigger and lead to systemic inflammatory responses. They can amplify the inflammatory cascade, and push the chemokines and other cytokines to release (Silva-Vaz et al., 2020). IL-6 is a marker of the severity of AP that mediated AP response (Escobar et al., 2012). Here, we measured the contents of TNF-*α*, IL-1 *β* and IL-6, and observed that miR-455-3p reduced their secretion levels in the cell culture supernatant.

In conclusion, miR-455-3p alleviated cell damage in AP cell model by regulating Slc2a1. This study offers a fresh idea for the exploration of AP treatment methods. However, there are also limitations. This study only briefly analyzed the expression of miR-455-3p/Slc2a at the animal level, and then conducted a mechanism study by performing cell experiments. The lack of further animal experiments and clinical trials is the biggest weakness of this study, which also arouses our enthusiasm to conduct follow-up research if there are sufficient time and funds. Meanwhile, due to time constraints, in-depth study of the potential role of other 4 hub genes of AP remains to be further conducted in the future.

##  Supplemental Information

10.7717/peerj.15612/supp-1 Supplemental Information 1 Raw data

10.7717/peerj.15612/supp-2 Supplemental Information 2 Author Checklist

## Additional Information and Declarations

### Data Availability

The following information was supplied regarding data availability: The data is available at NCBI GEO: GSE81260, GSE109227 and GSE161945.
