# Supplementary material for: miR-455-3p ameliorates pancreatic acinar cell injury by targeting Slc2a1
Source: PeerJ. 2023 Jun 30;11:e15612. doi: 10.7717/peerj.15612 (PMC10317017; doi:10.7717/peerj.15612)
Supplement: Supplemental Information 1 [file peerj-11-15612-s001.zip › Raw data/Fig1.2.3/Bioinformatics.pptx]

## Slide 1
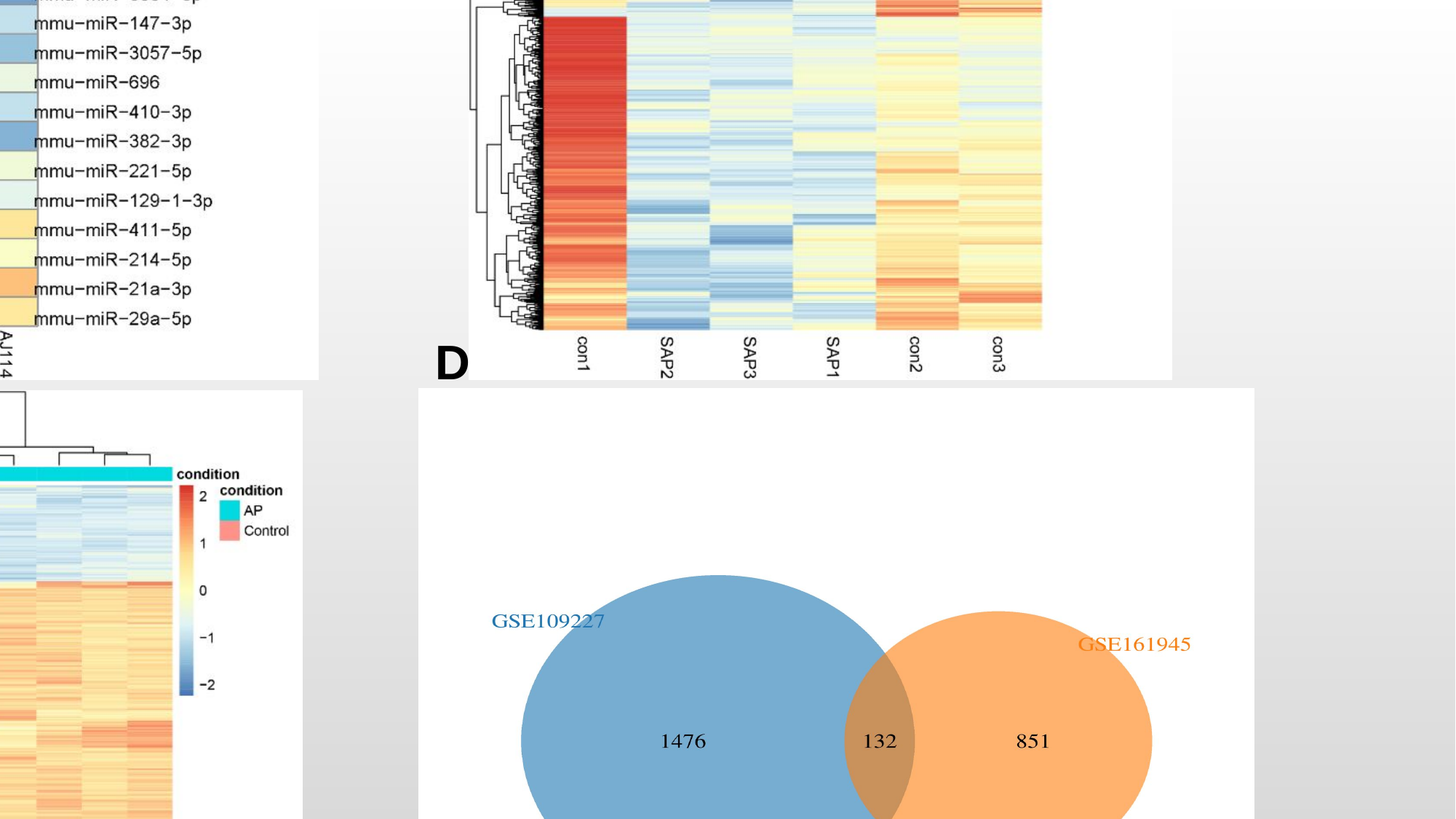

A
B
C
D

## Slide 2
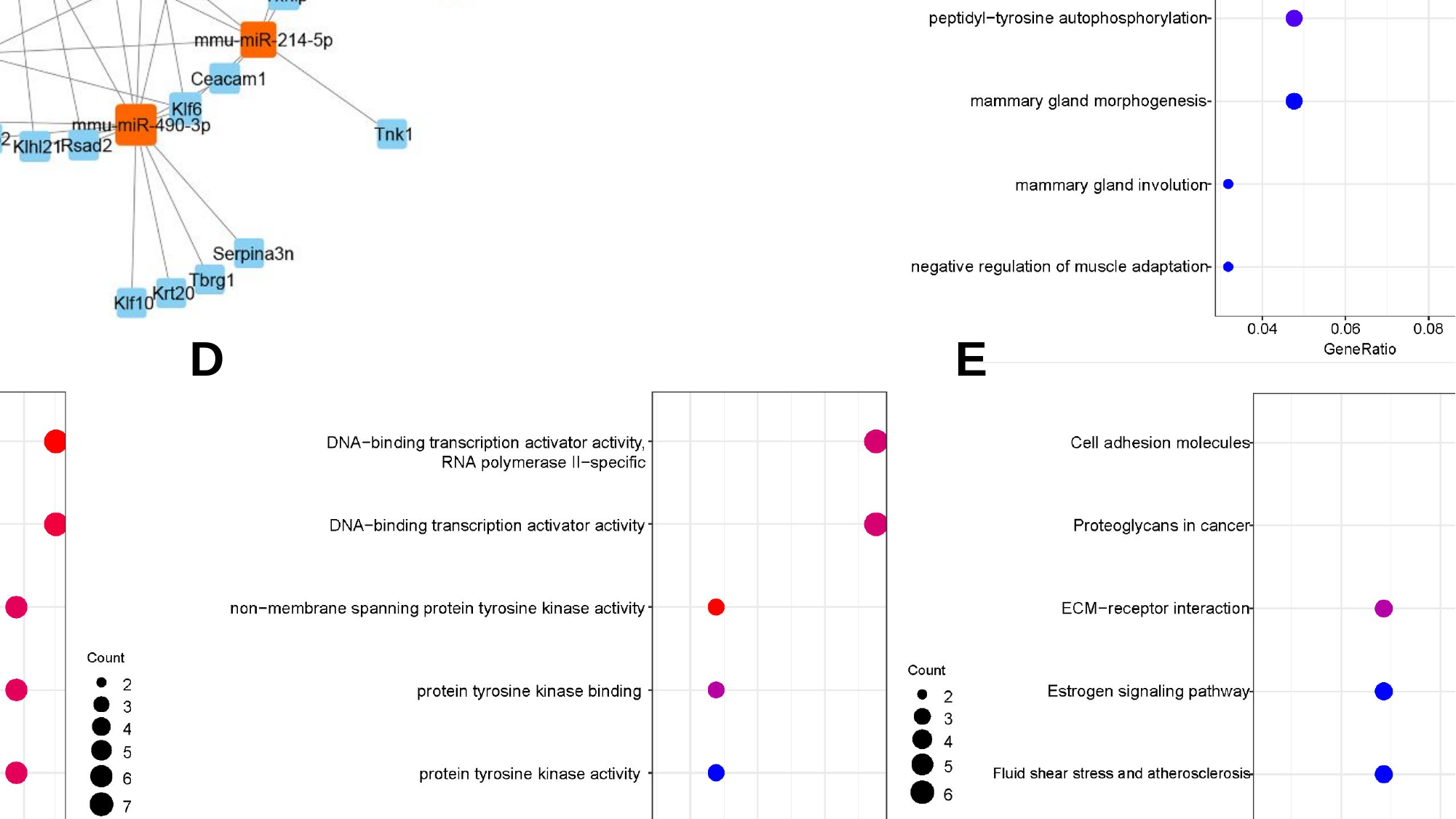

A
B
C
D
E
图2 靶基因的筛选及功能富集分析。
A：miRNA-mRNA 调控网络；B-E：GO-BP、GO-CC、GO-MF、 KEGG对miRNA的靶基因进行功能富集分析。

## Slide 3
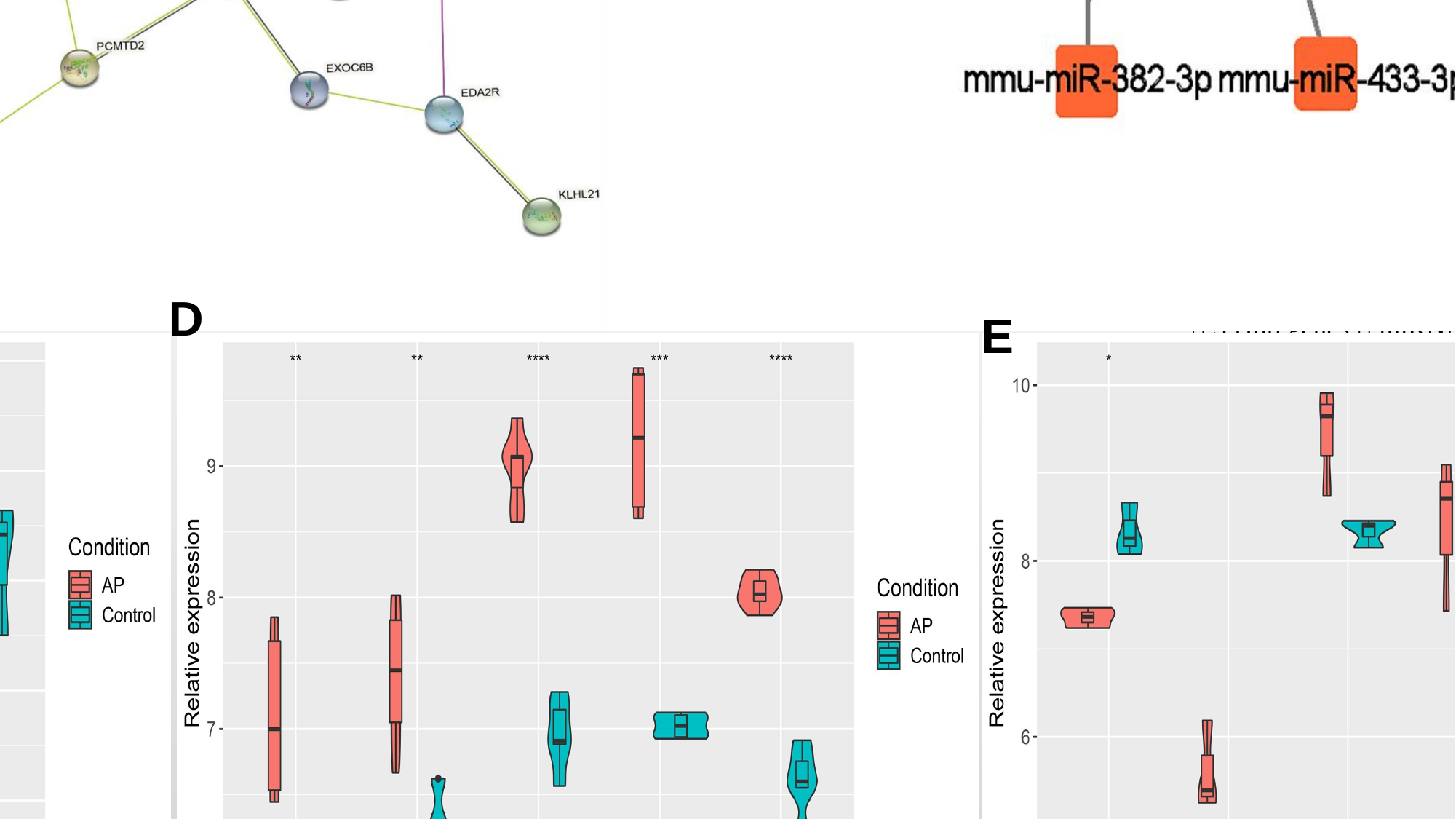

A
B
C
D
E
图3 Hub genes与miRNA的调控关系。
A：PPI网络；B：miRNA和hub genes的调控关系；C：AP和Control组中调控hub genes的miRNA的表达；D-E：GSE109227和GSE161945中hub genes的表达。
