# Supplementary figures and images for: miR-455-3p ameliorates pancreatic acinar cell injury by targeting Slc2a1
Source: PeerJ. 2023 Jun 30;11:e15612. doi: 10.7717/peerj.15612 (PMC10317017; doi:10.7717/peerj.15612)

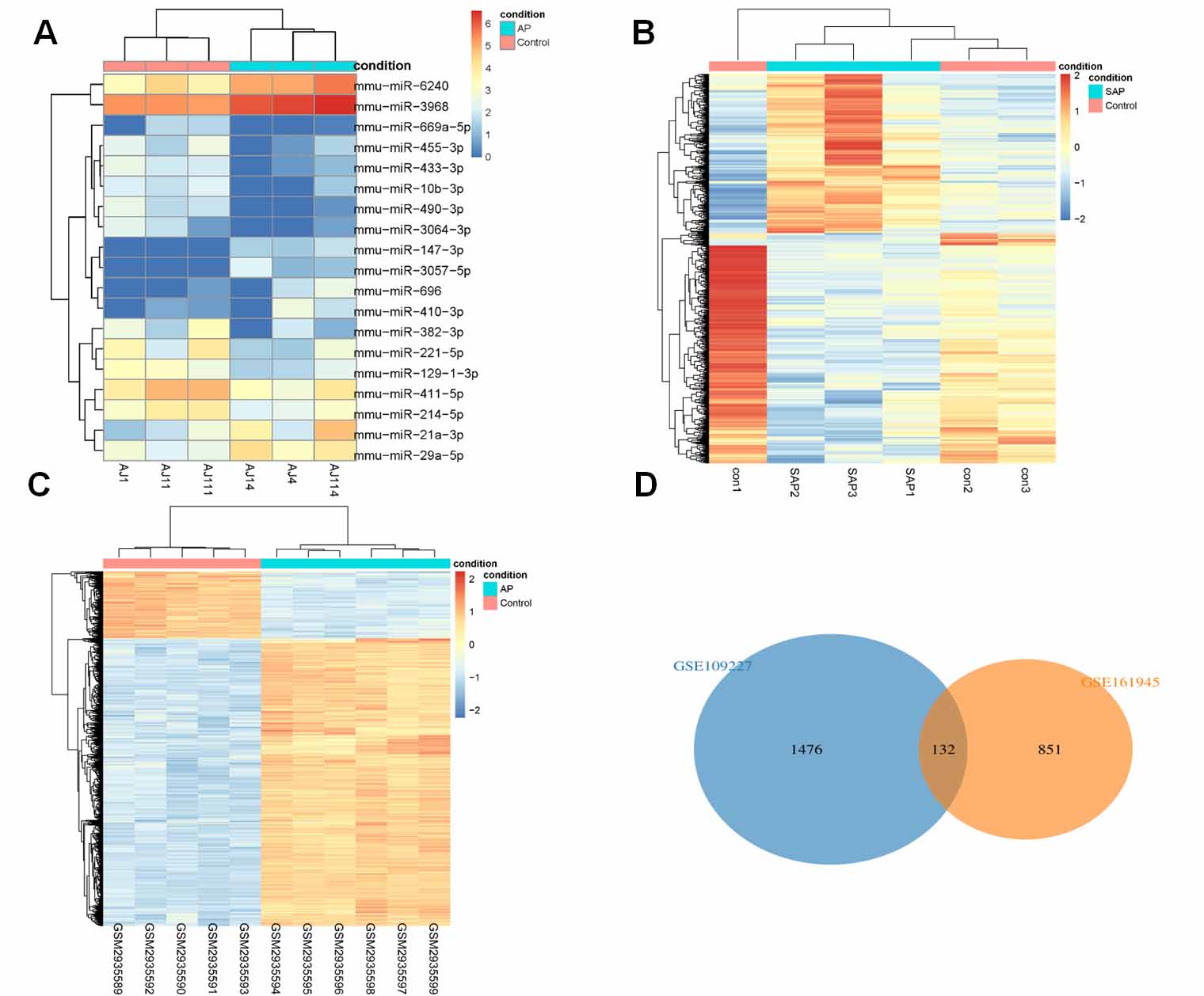

Supplement: Supplemental Information 1 [file peerj-11-15612-s001.zip › Raw data/Fig1.2.3/Bioinformatics 1.jpg]

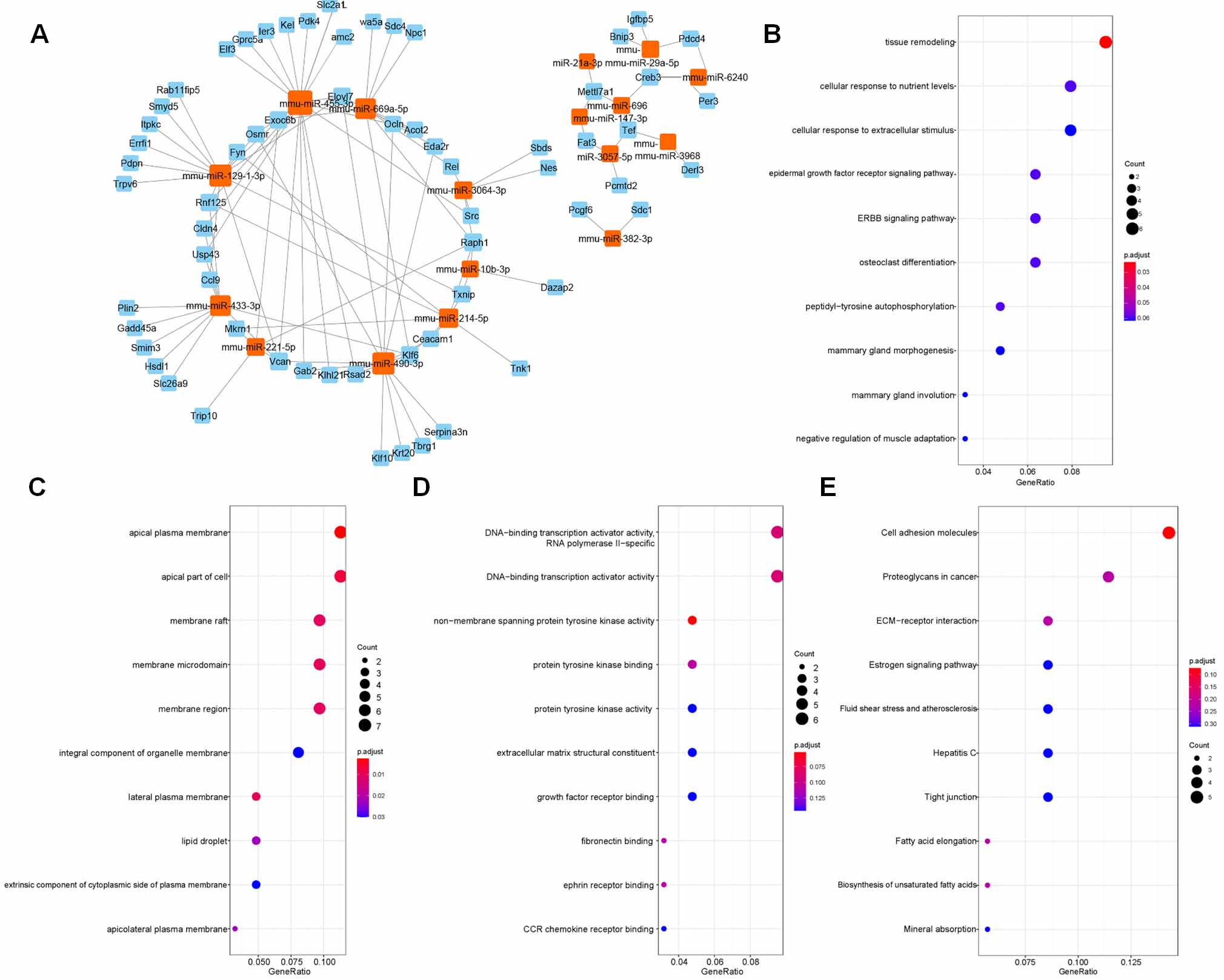

Supplement: Supplemental Information 1 [file peerj-11-15612-s001.zip › Raw data/Fig1.2.3/Bioinformatics 2.jpg]

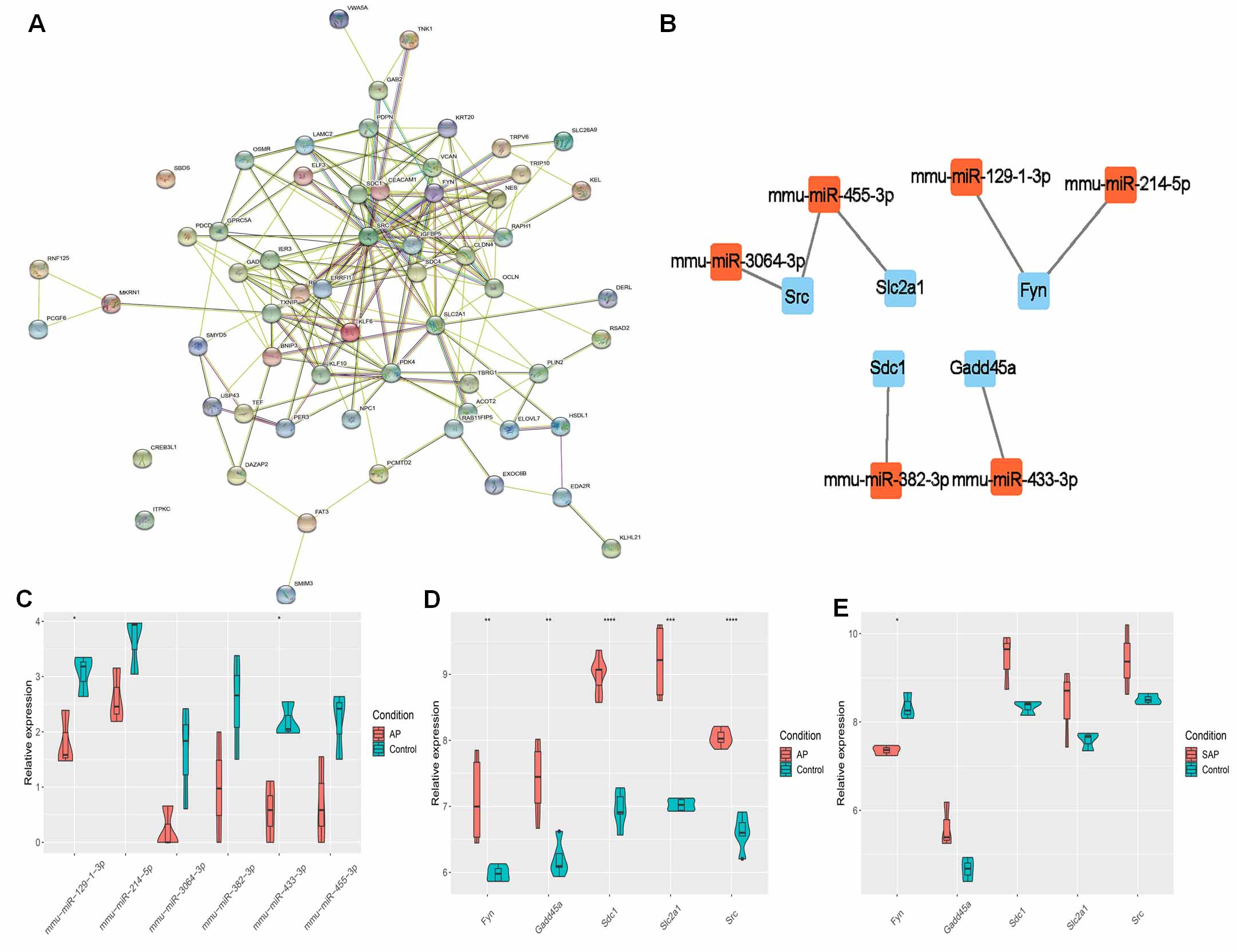

Supplement: Supplemental Information 1 [file peerj-11-15612-s001.zip › Raw data/Fig1.2.3/Bioinformatics 3.jpg]

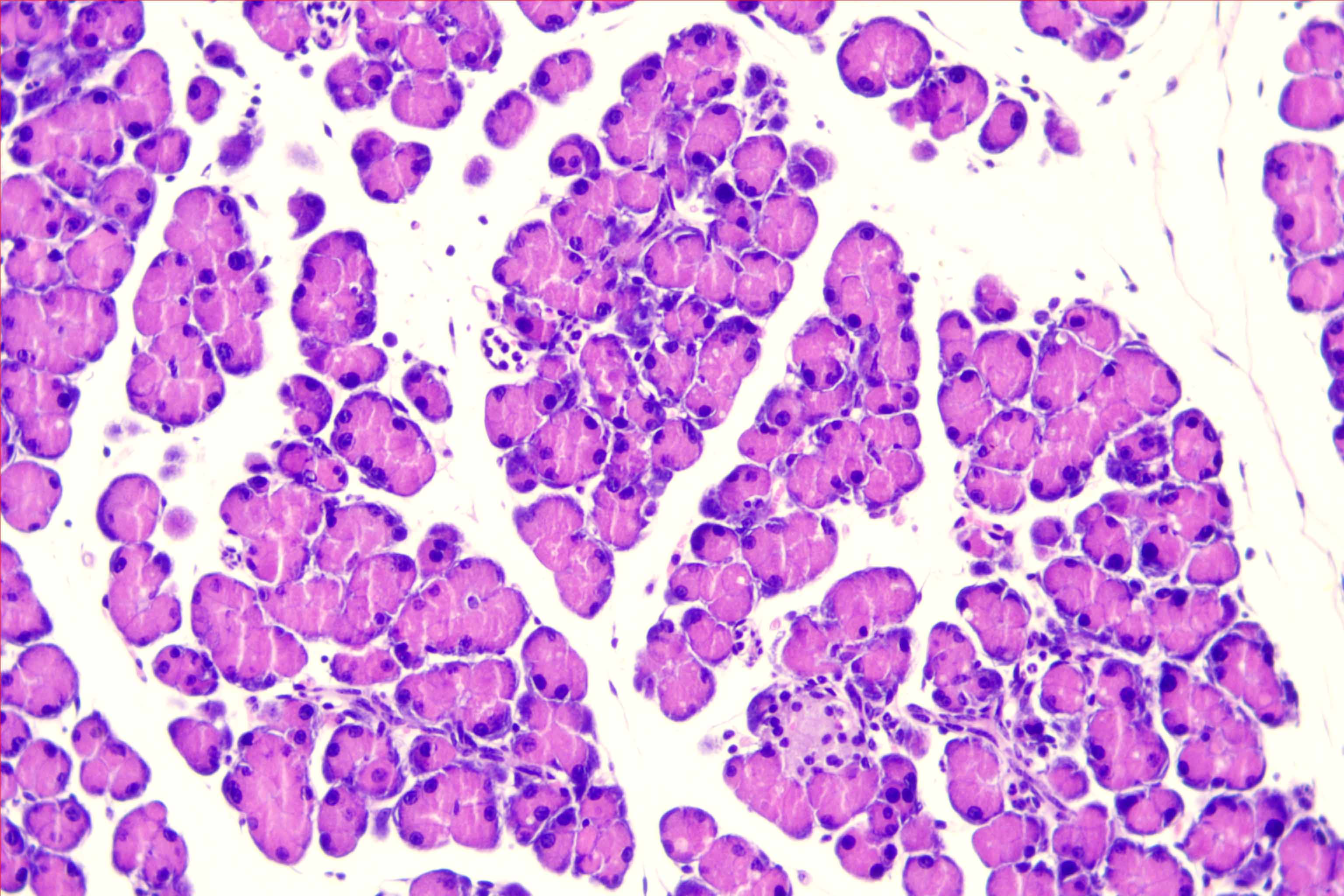

Supplement: Supplemental Information 1 [file peerj-11-15612-s001.zip › Raw data/HE staining/AP.jpg]

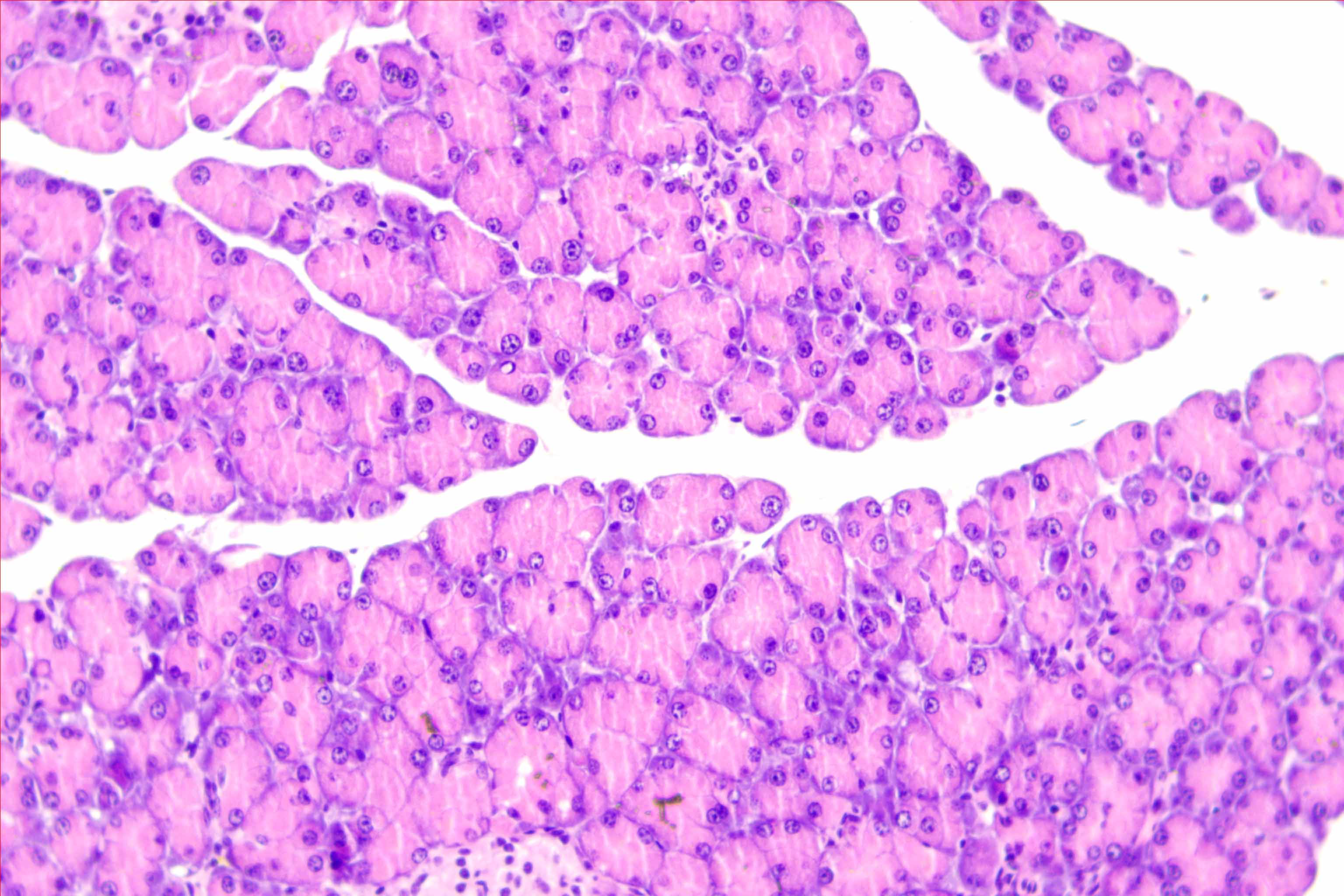

Supplement: Supplemental Information 1 [file peerj-11-15612-s001.zip › Raw data/HE staining/Control.jpg]

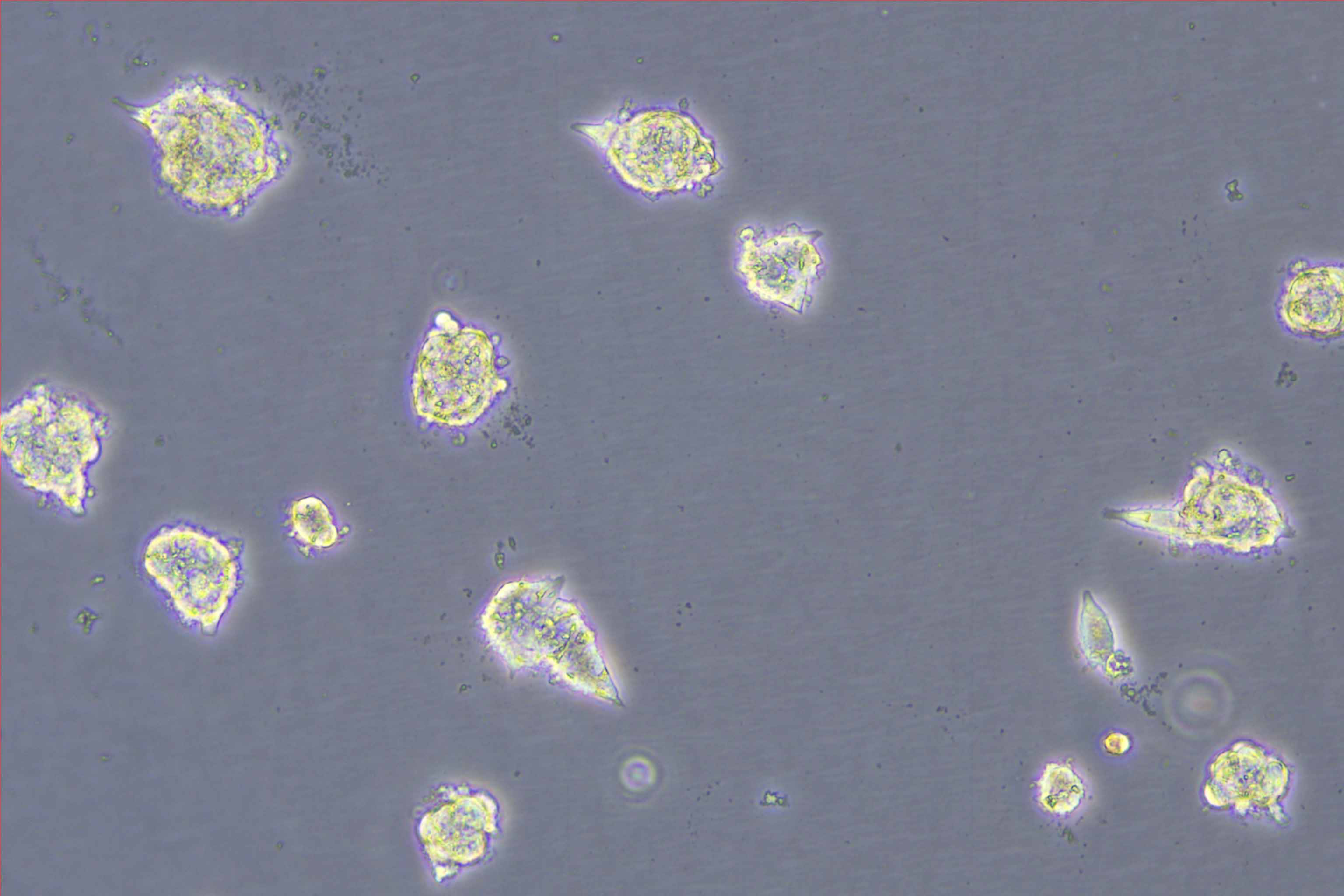

Supplement: Supplemental Information 1 [file peerj-11-15612-s001.zip › Raw data/Morphological Observation/Figure 6C/AP+miR-455-3p mimics.jpg]

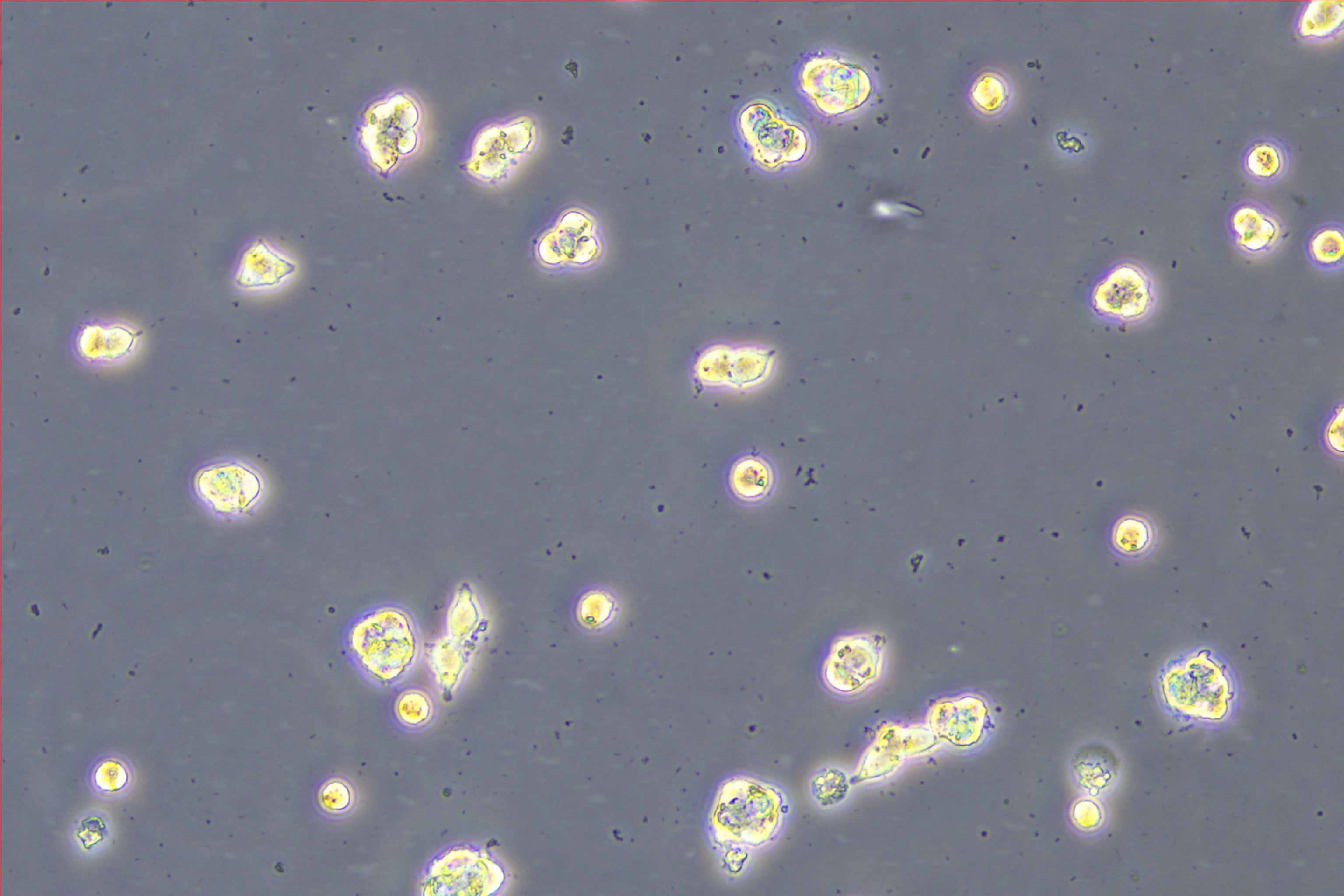

Supplement: Supplemental Information 1 [file peerj-11-15612-s001.zip › Raw data/Morphological Observation/Figure 6C/AP+miRNA-NC.jpg]

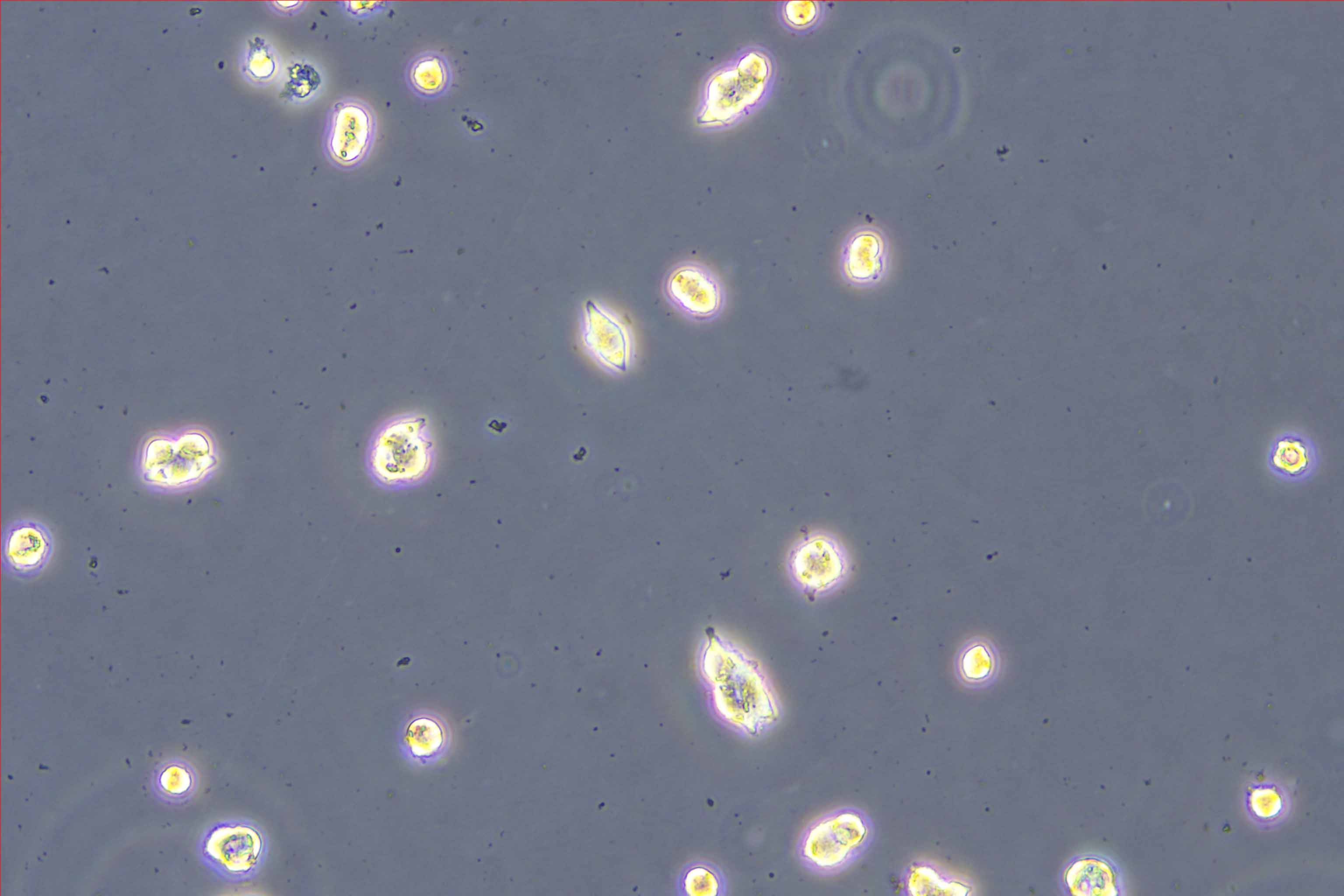

Supplement: Supplemental Information 1 [file peerj-11-15612-s001.zip › Raw data/Morphological Observation/Figure 6C/AP.jpg]

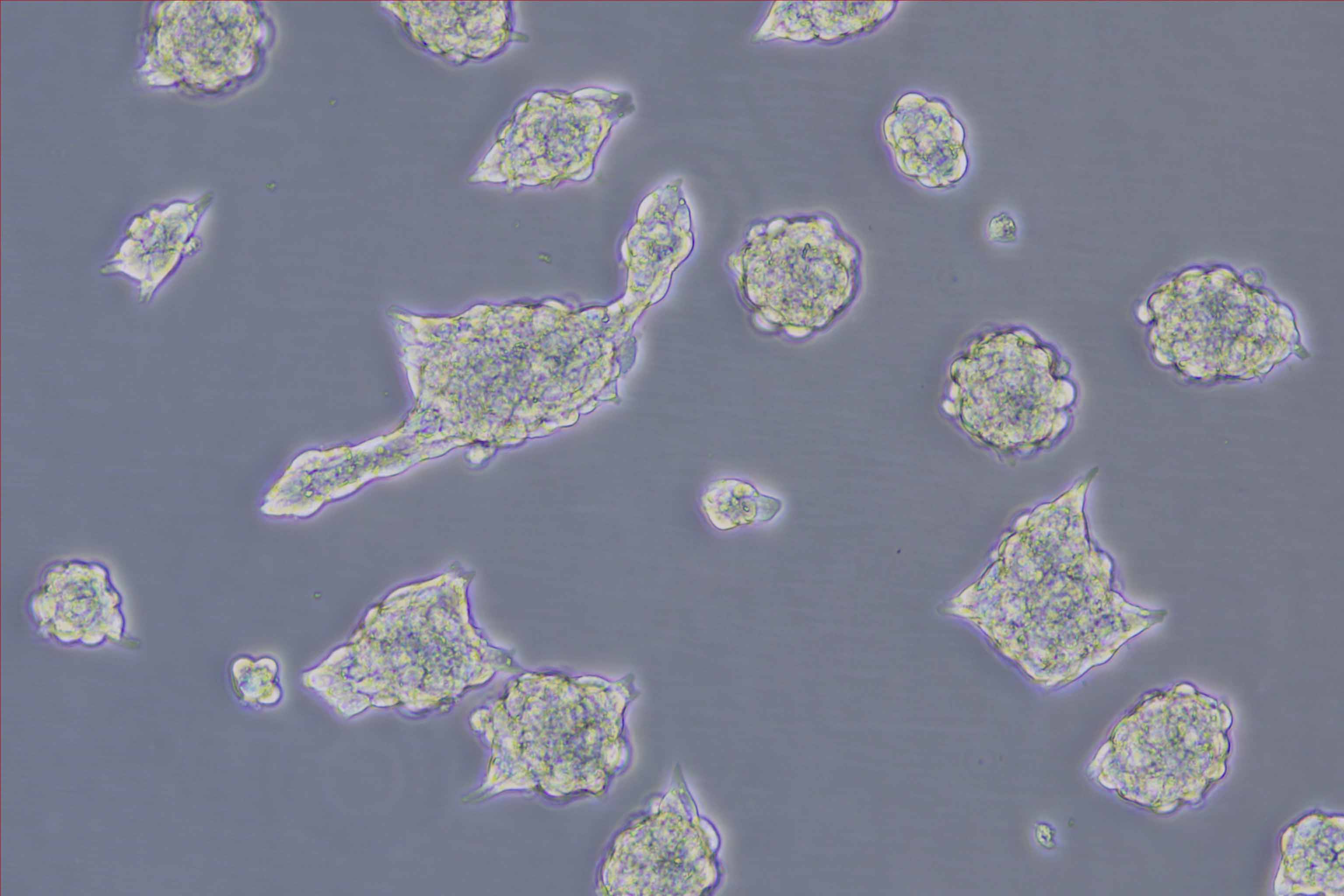

Supplement: Supplemental Information 1 [file peerj-11-15612-s001.zip › Raw data/Morphological Observation/Figure 6C/Control.jpg]

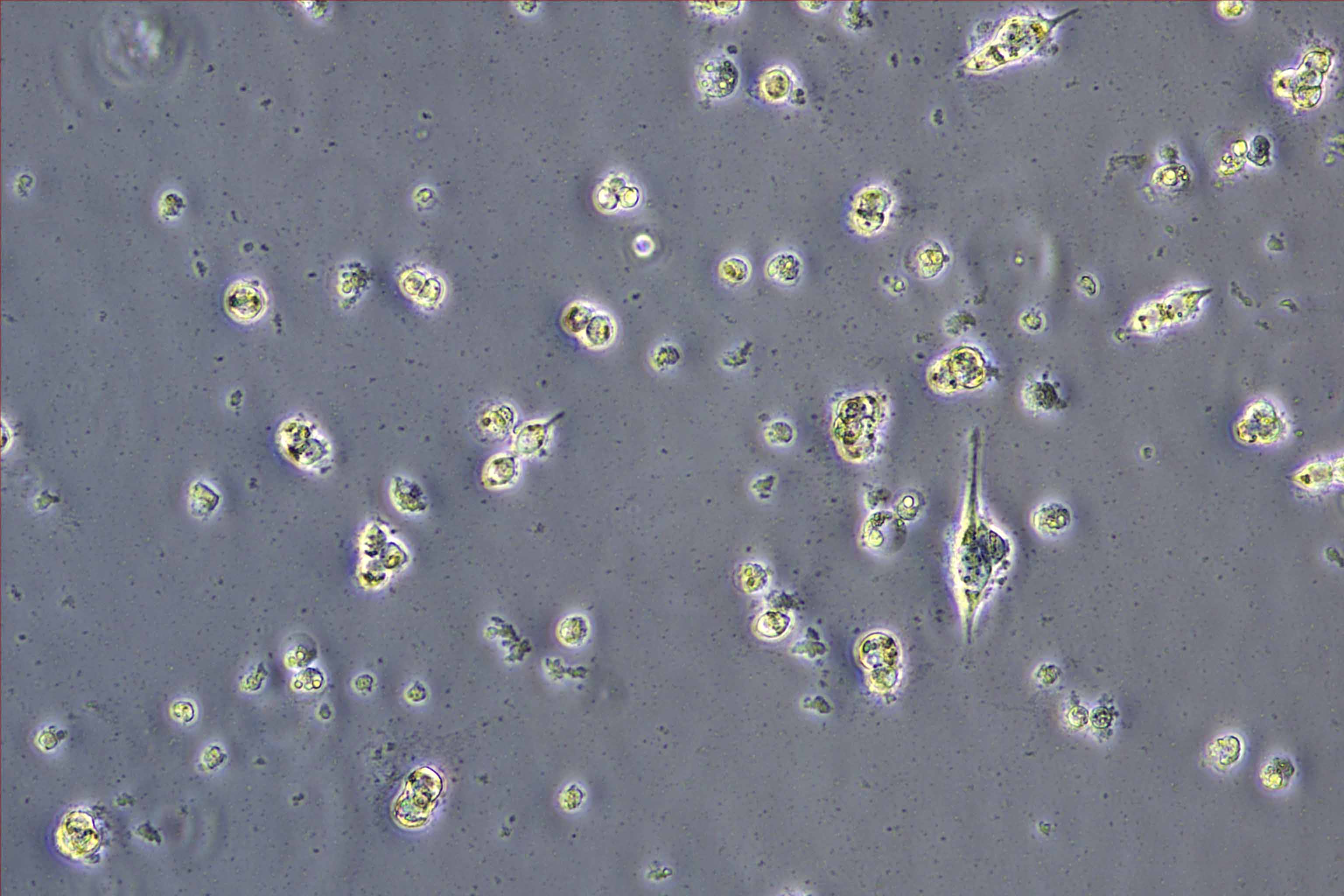

Supplement: Supplemental Information 1 [file peerj-11-15612-s001.zip › Raw data/Morphological Observation/Figure 8C/AP+miR-455-3p inhibitor+siSlc2a1.jpg]

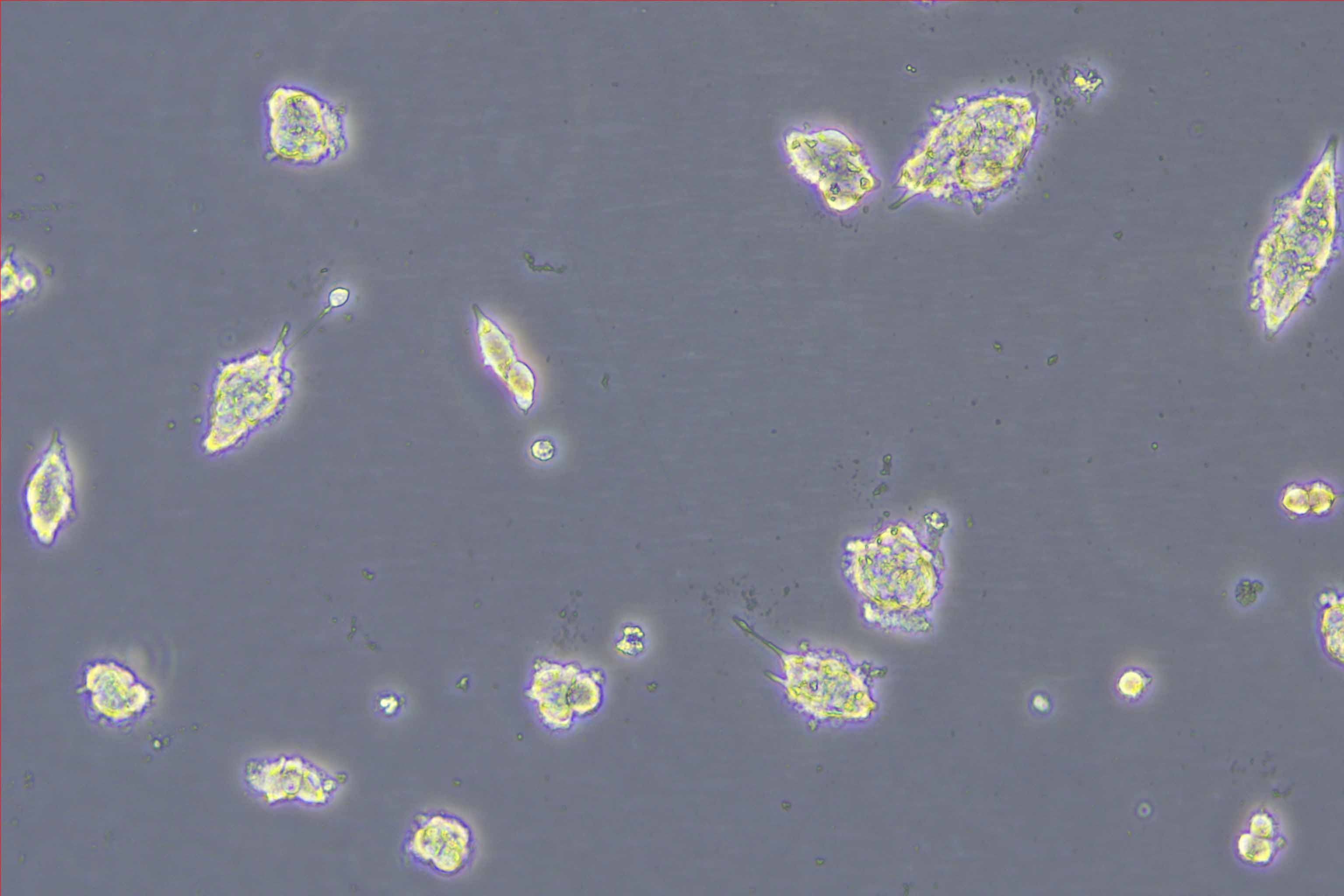

Supplement: Supplemental Information 1 [file peerj-11-15612-s001.zip › Raw data/Morphological Observation/Figure 8C/AP+miRNA-NC+siSlc2a1.jpg]

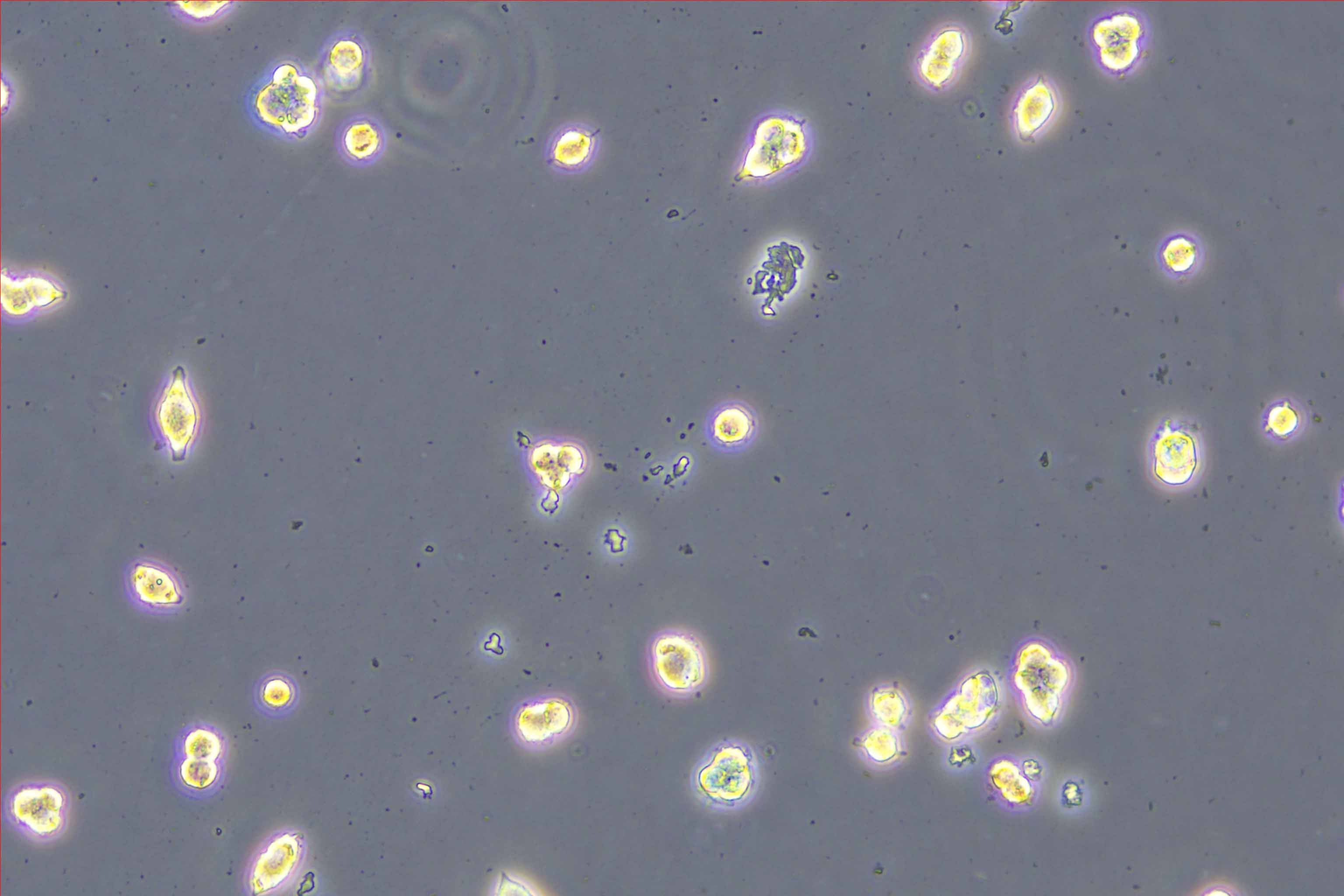

Supplement: Supplemental Information 1 [file peerj-11-15612-s001.zip › Raw data/Morphological Observation/Figure 8C/AP+siNC.jpg]

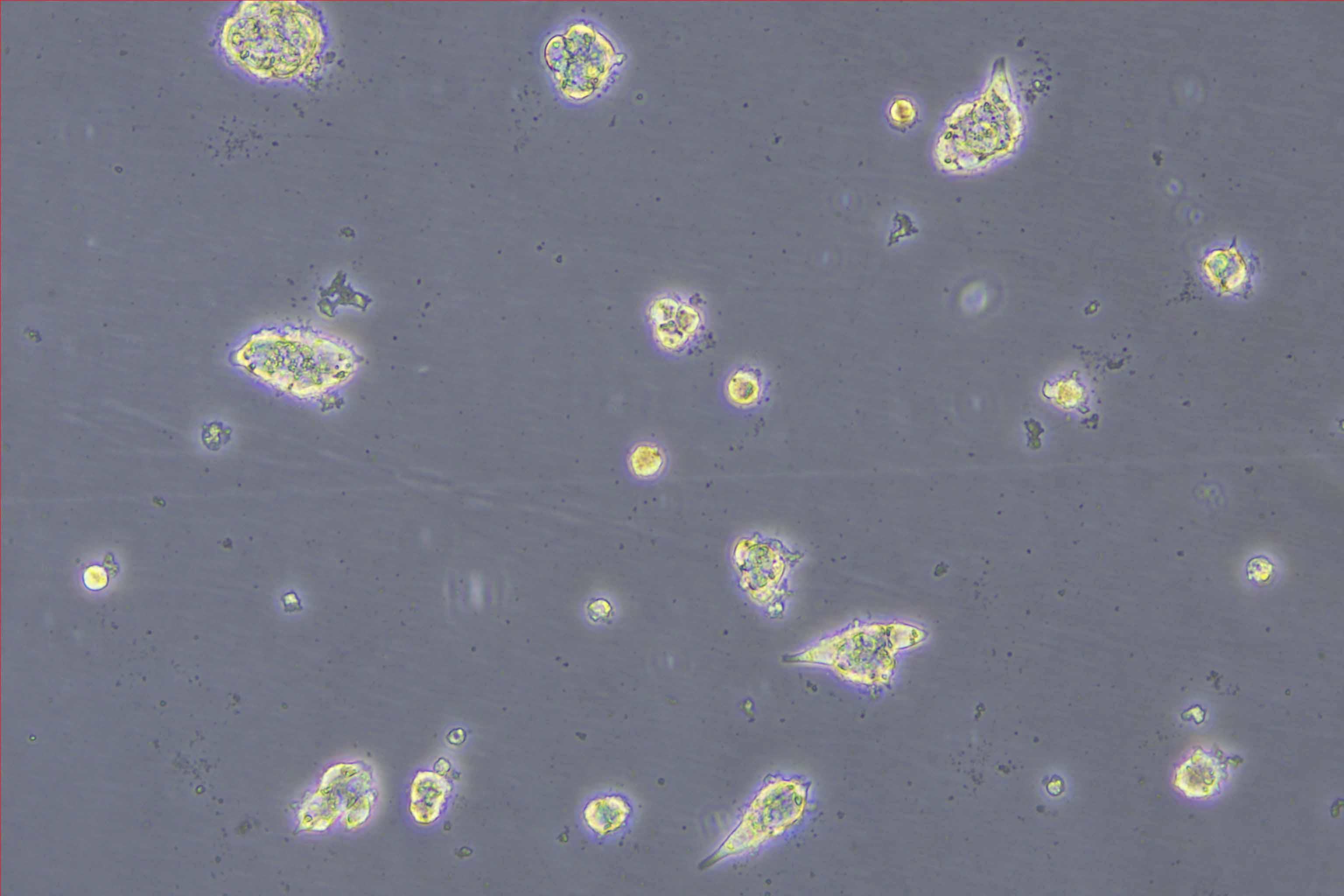

Supplement: Supplemental Information 1 [file peerj-11-15612-s001.zip › Raw data/Morphological Observation/Figure 8C/AP+siSlc2a1.jpg]

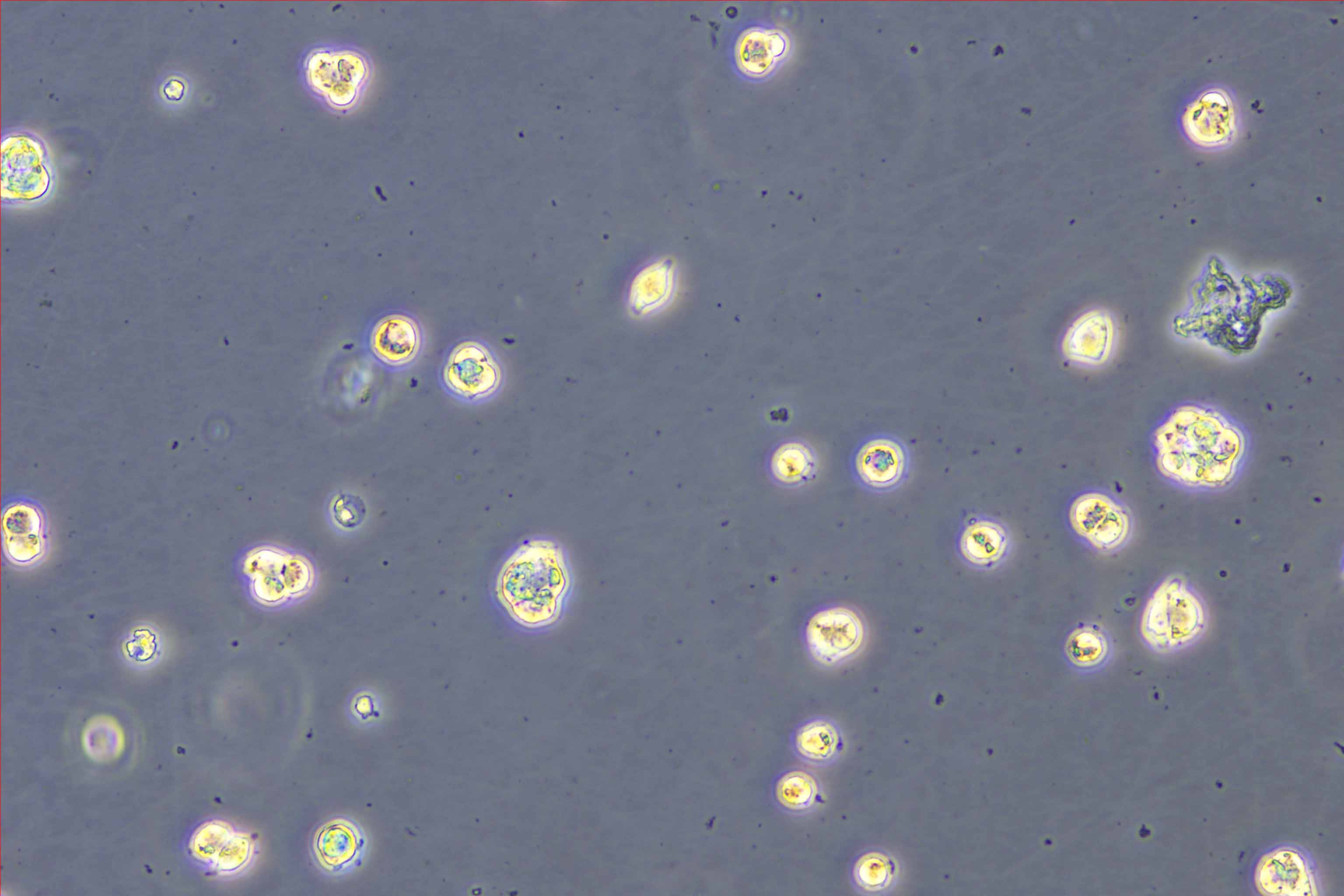

Supplement: Supplemental Information 1 [file peerj-11-15612-s001.zip › Raw data/Morphological Observation/Figure 8C/AP.jpg]

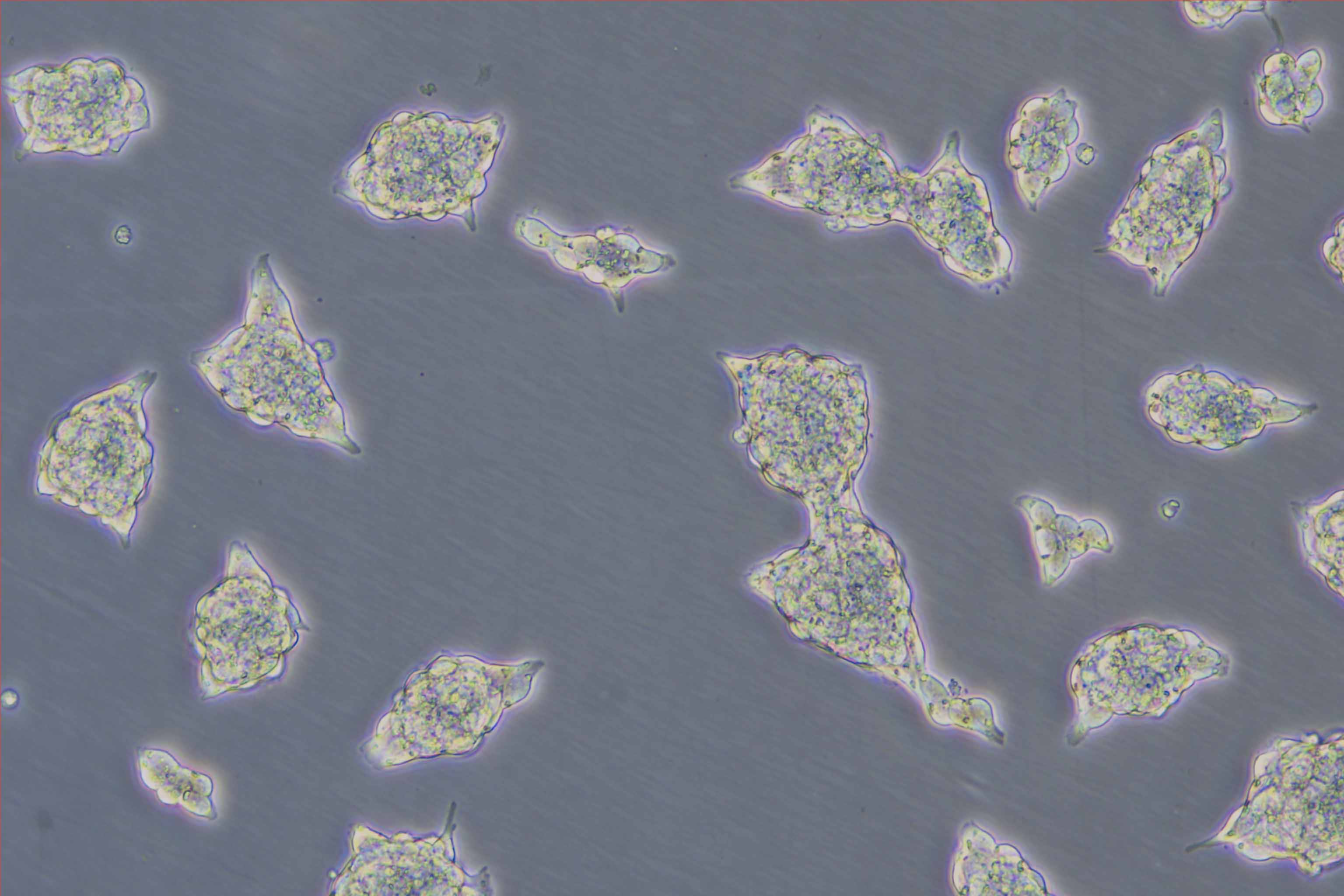

Supplement: Supplemental Information 1 [file peerj-11-15612-s001.zip › Raw data/Morphological Observation/Figure 8C/Control.jpg]

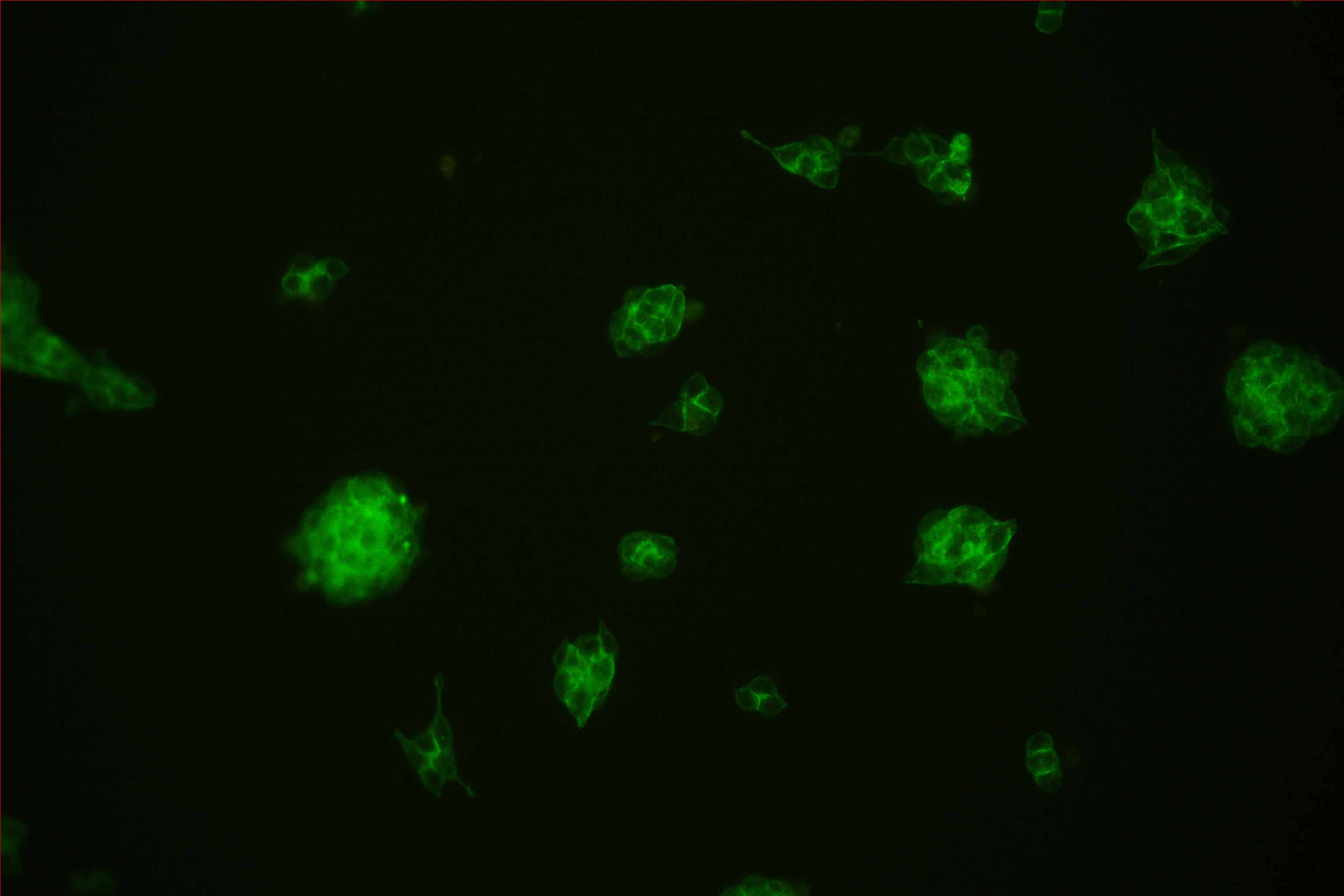

Supplement: Supplemental Information 1 [file peerj-11-15612-s001.zip › Raw data/Trypsin activity(Rhodamine 110)/Figure 6D/AP+miR-455-3p mimics.jpg]

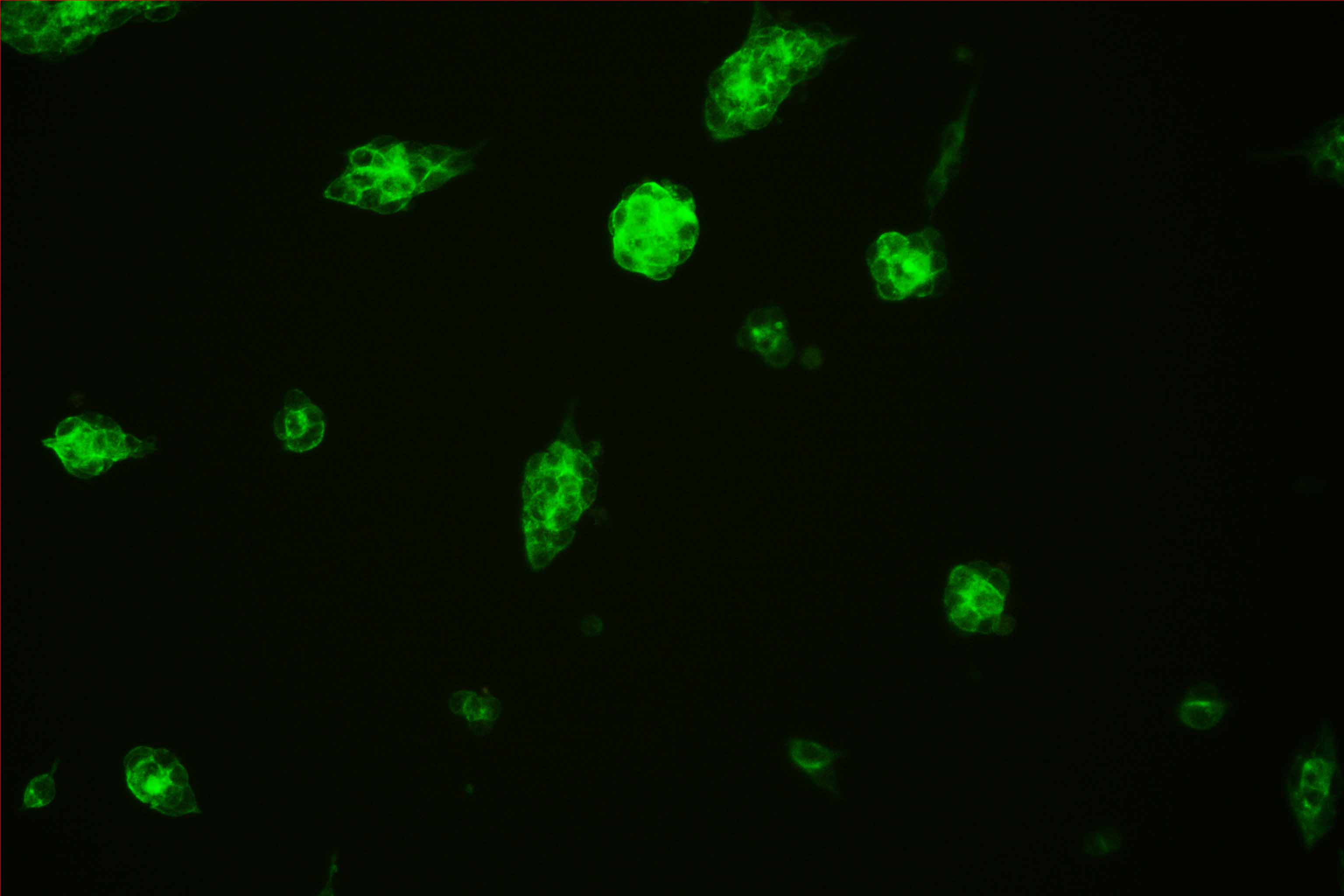

Supplement: Supplemental Information 1 [file peerj-11-15612-s001.zip › Raw data/Trypsin activity(Rhodamine 110)/Figure 6D/AP+miRNA-NC.jpg]

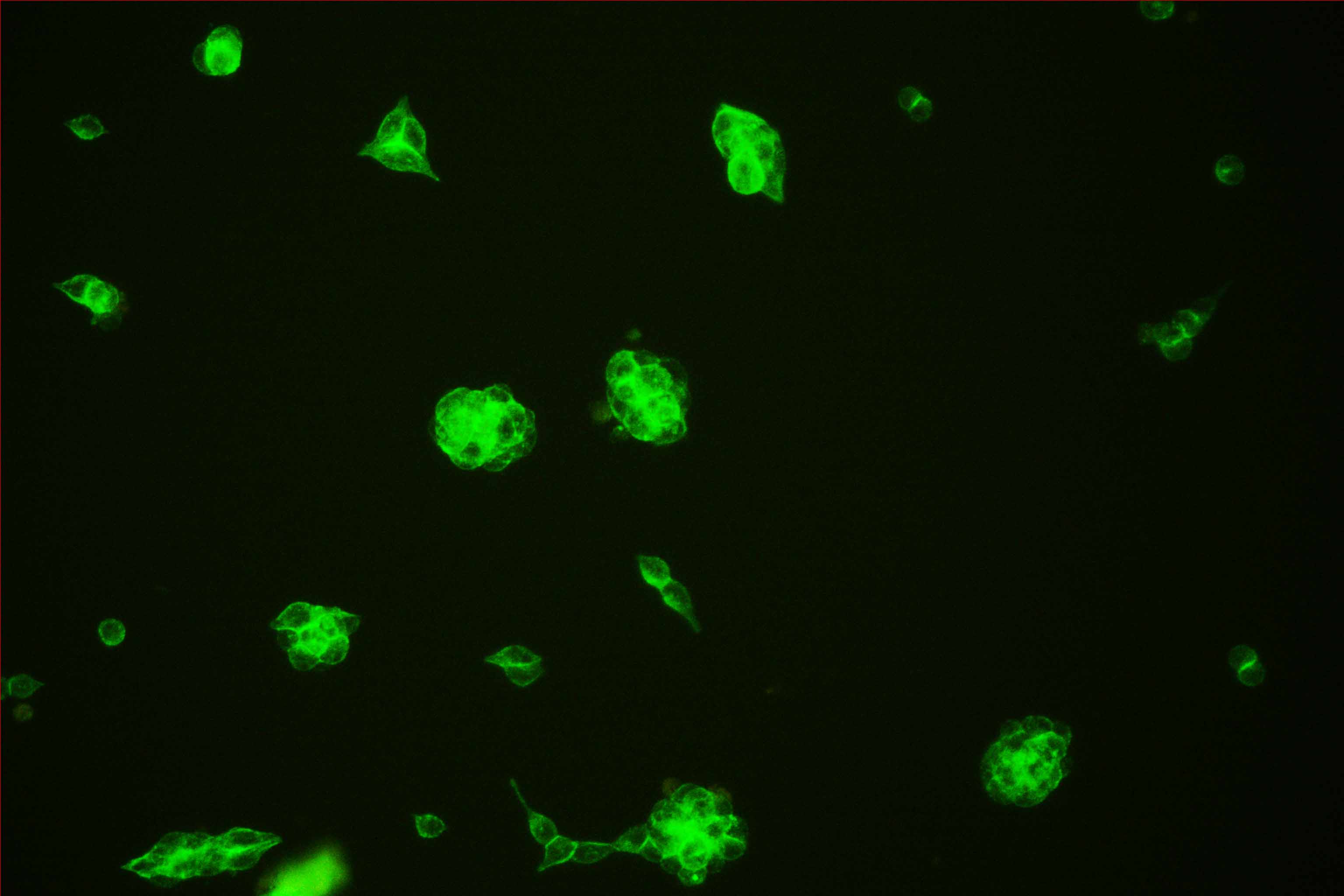

Supplement: Supplemental Information 1 [file peerj-11-15612-s001.zip › Raw data/Trypsin activity(Rhodamine 110)/Figure 6D/AP.jpg]

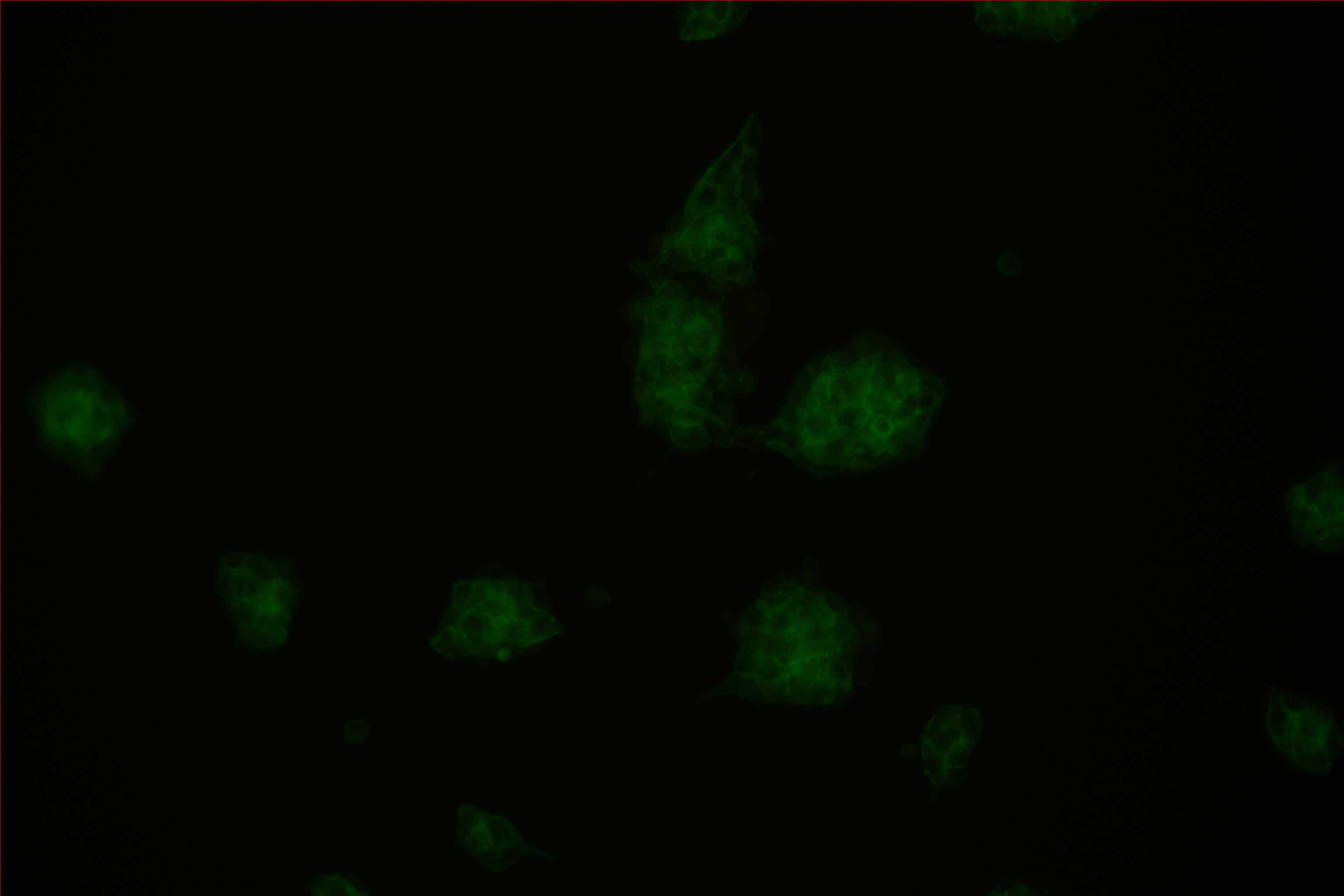

Supplement: Supplemental Information 1 [file peerj-11-15612-s001.zip › Raw data/Trypsin activity(Rhodamine 110)/Figure 6D/Control.jpg]

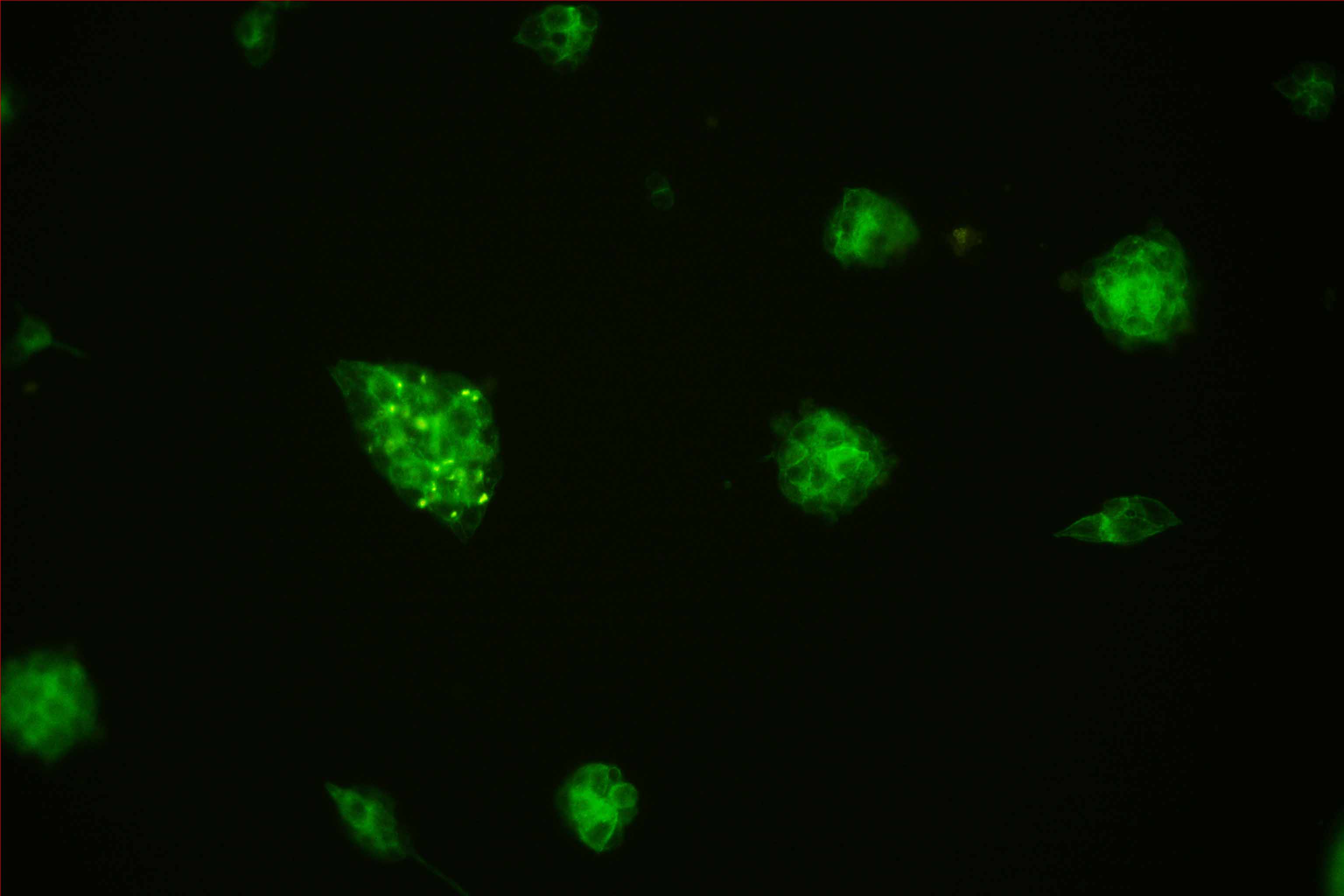

Supplement: Supplemental Information 1 [file peerj-11-15612-s001.zip › Raw data/Trypsin activity(Rhodamine 110)/Figure 8D/AP+miR-455-3p inhibitor+siSlc2a1.jpg]

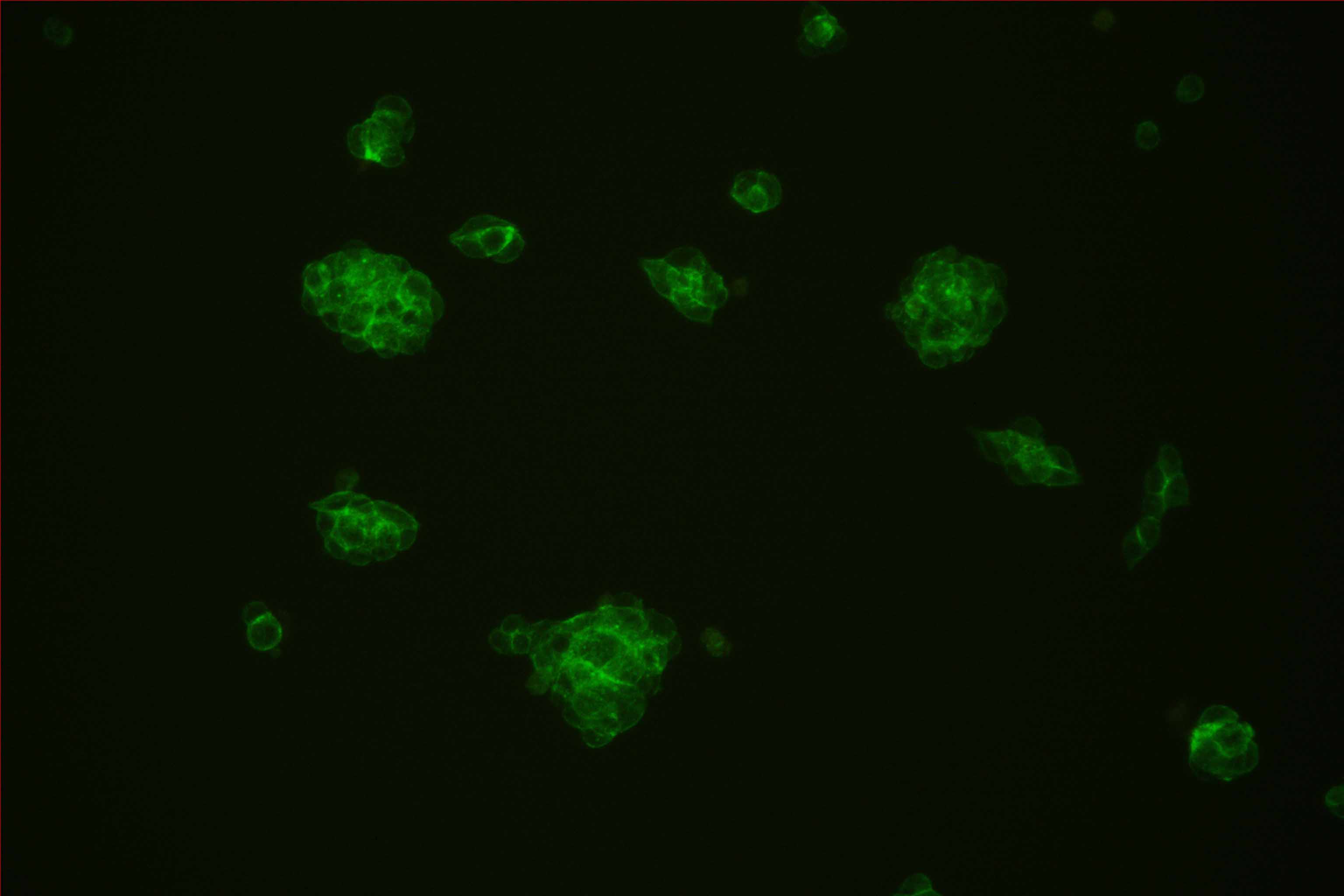

Supplement: Supplemental Information 1 [file peerj-11-15612-s001.zip › Raw data/Trypsin activity(Rhodamine 110)/Figure 8D/AP+miRNA-NC+siSlc2a1.jpg]

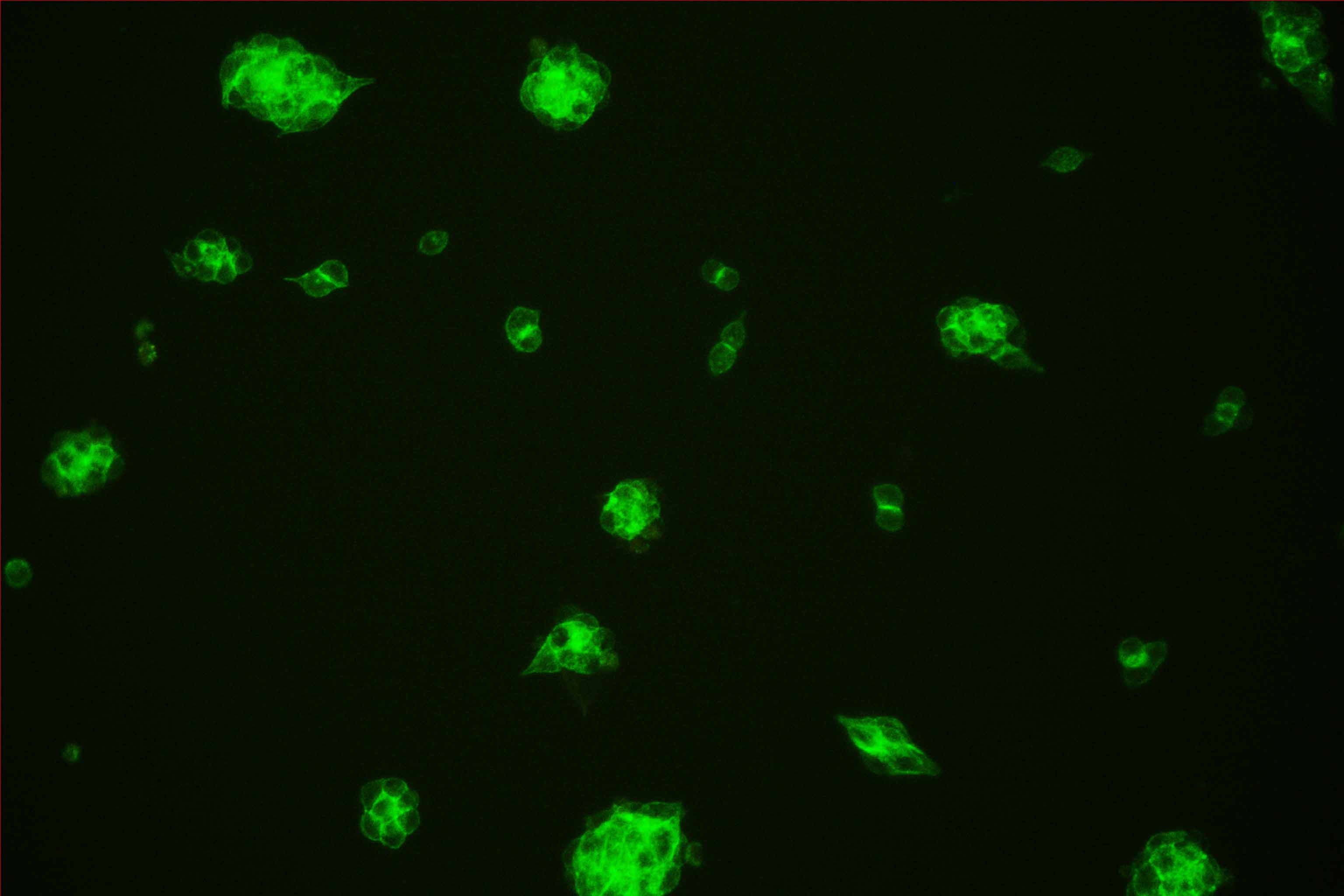

Supplement: Supplemental Information 1 [file peerj-11-15612-s001.zip › Raw data/Trypsin activity(Rhodamine 110)/Figure 8D/AP+siNC.jpg]

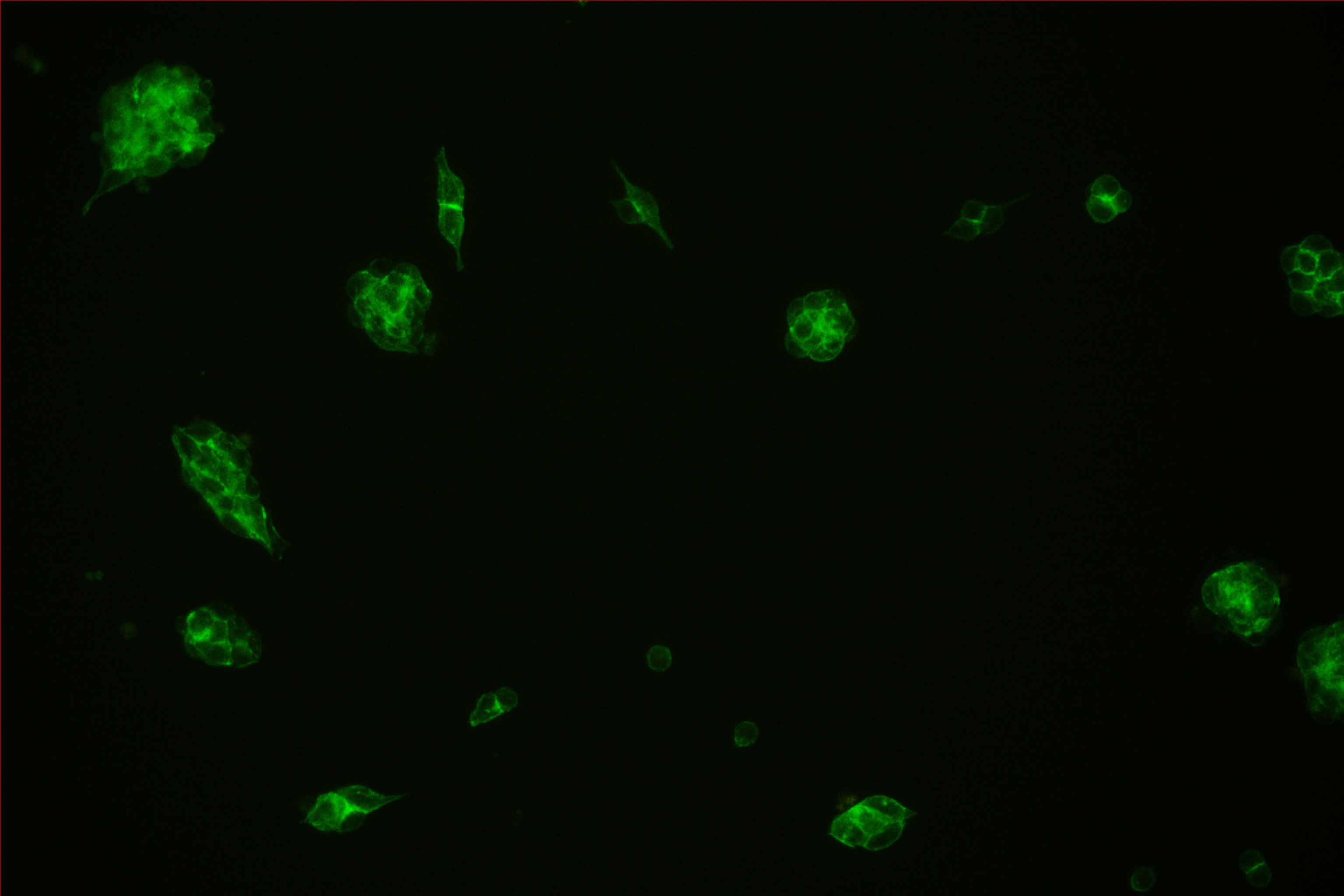

Supplement: Supplemental Information 1 [file peerj-11-15612-s001.zip › Raw data/Trypsin activity(Rhodamine 110)/Figure 8D/AP+siSlc2a1.jpg]

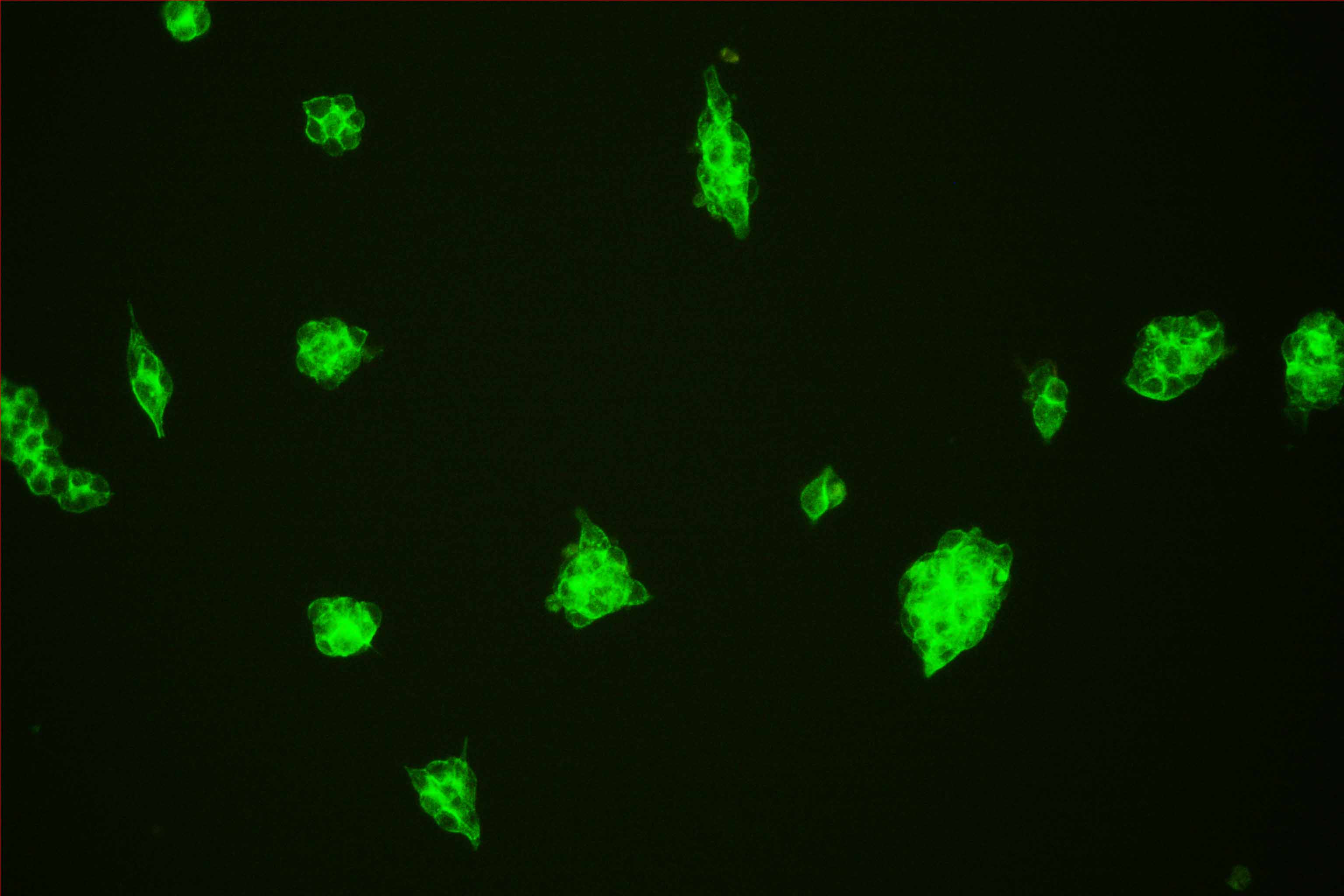

Supplement: Supplemental Information 1 [file peerj-11-15612-s001.zip › Raw data/Trypsin activity(Rhodamine 110)/Figure 8D/AP.jpg]

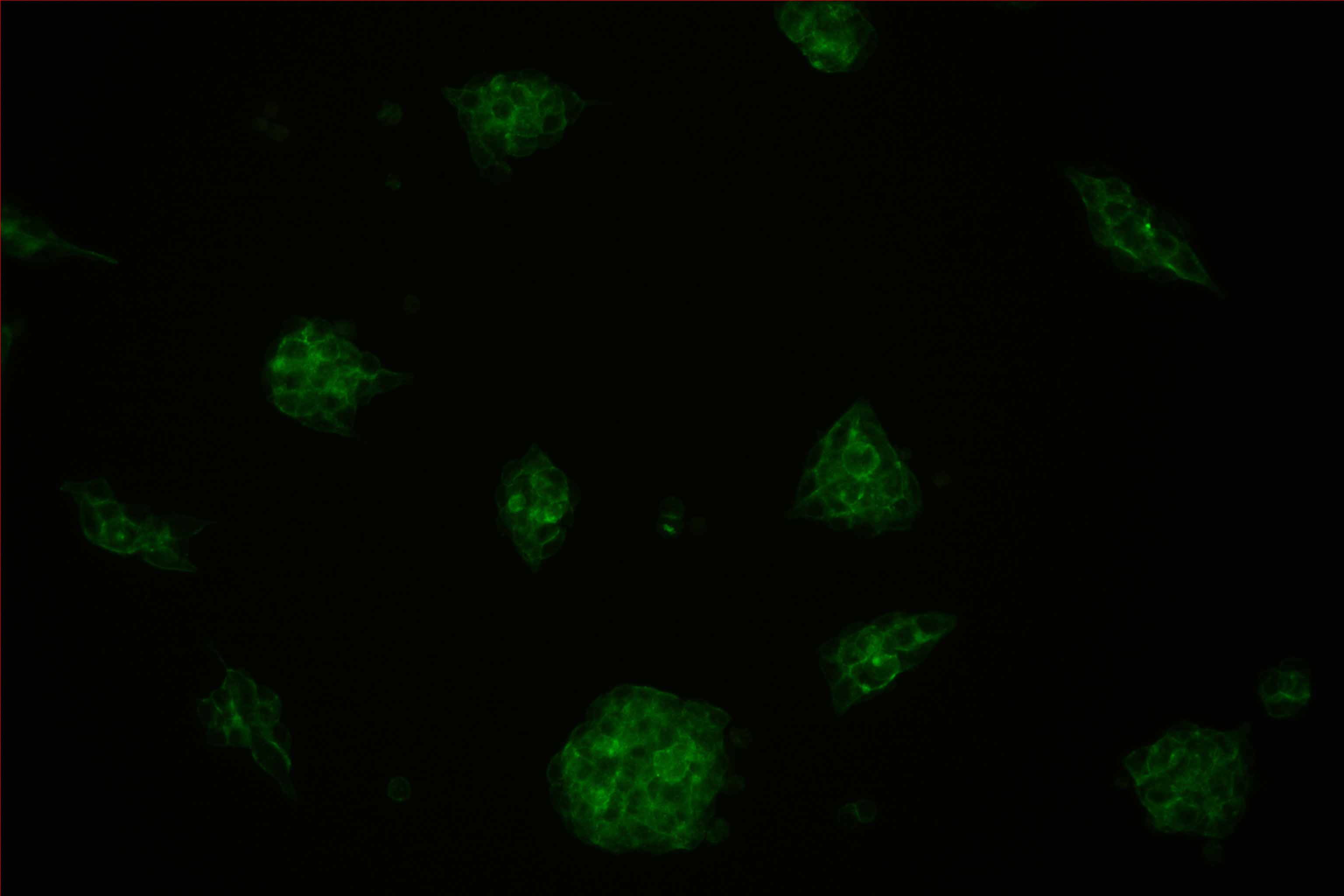

Supplement: Supplemental Information 1 [file peerj-11-15612-s001.zip › Raw data/Trypsin activity(Rhodamine 110)/Figure 8D/Control.jpg]

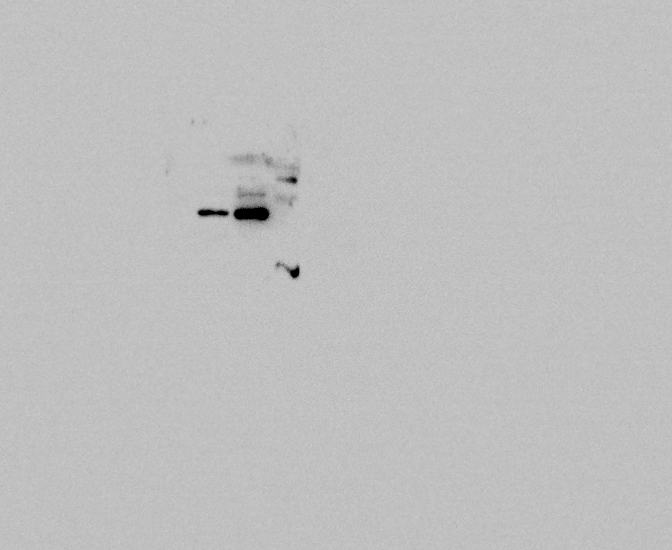

Supplement: Supplemental Information 1 [file peerj-11-15612-s001.zip › Raw data/Western Blot/5B/Slc2a1 (1).tif]

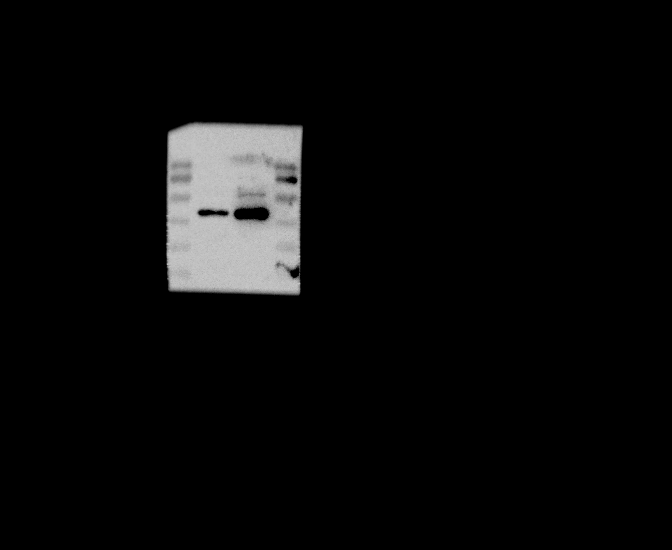

Supplement: Supplemental Information 1 [file peerj-11-15612-s001.zip › Raw data/Western Blot/5B/Slc2a1 (2).tif]

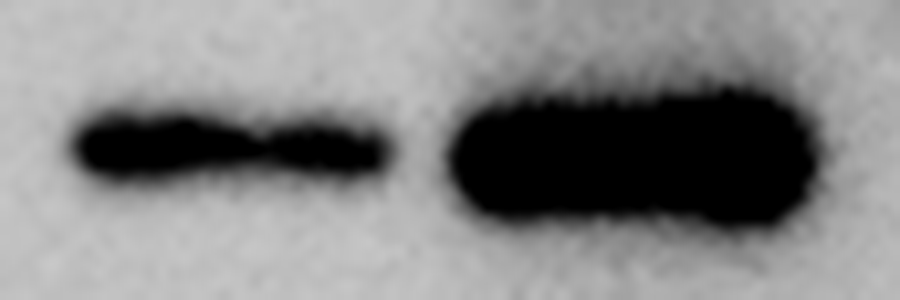

Supplement: Supplemental Information 1 [file peerj-11-15612-s001.zip › Raw data/Western Blot/5B/Slc2a1 (3).tif]

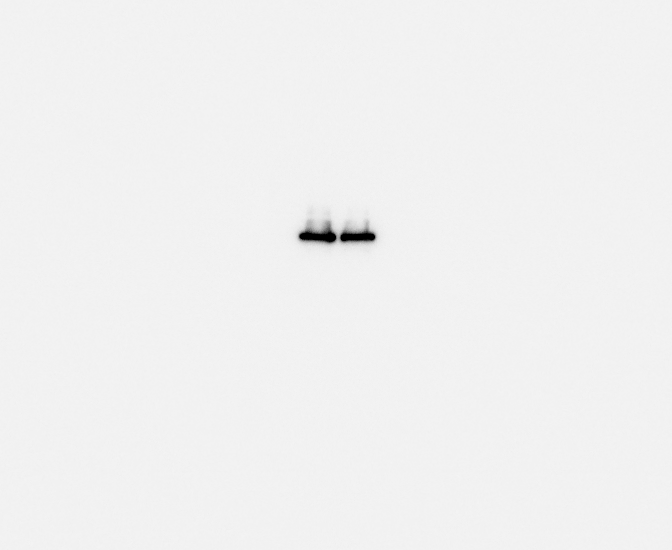

Supplement: Supplemental Information 1 [file peerj-11-15612-s001.zip › Raw data/Western Blot/5B/β-actin (1).tif]

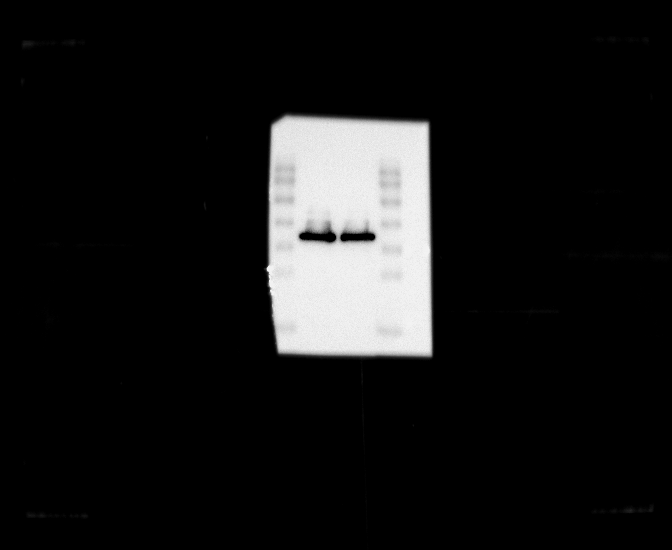

Supplement: Supplemental Information 1 [file peerj-11-15612-s001.zip › Raw data/Western Blot/5B/β-actin (2).tif]

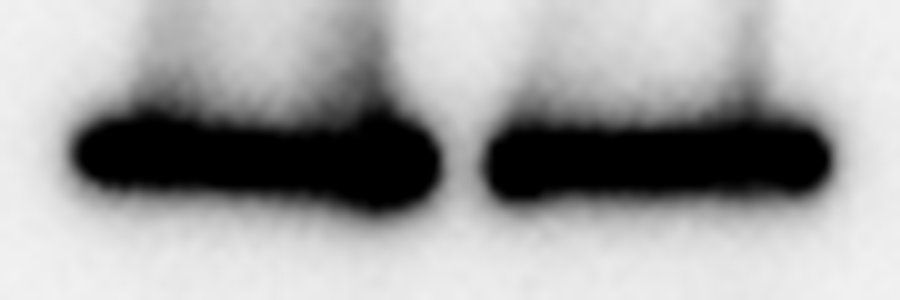

Supplement: Supplemental Information 1 [file peerj-11-15612-s001.zip › Raw data/Western Blot/5B/β-actin (3).tif]

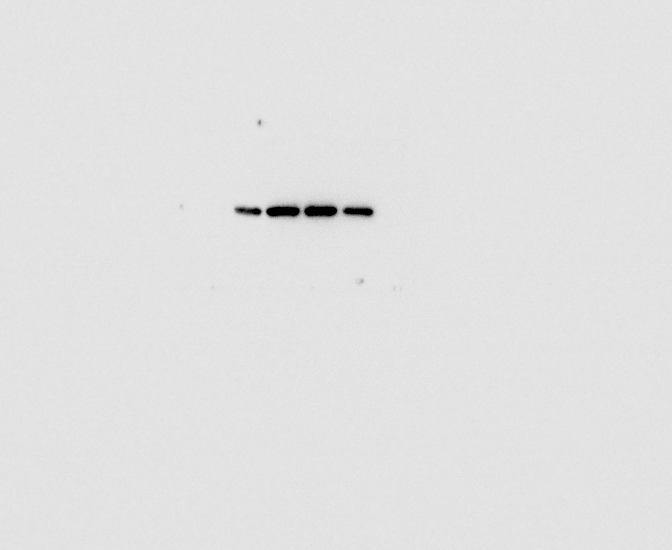

Supplement: Supplemental Information 1 [file peerj-11-15612-s001.zip › Raw data/Western Blot/6B/Slc2a1 (1).tif]

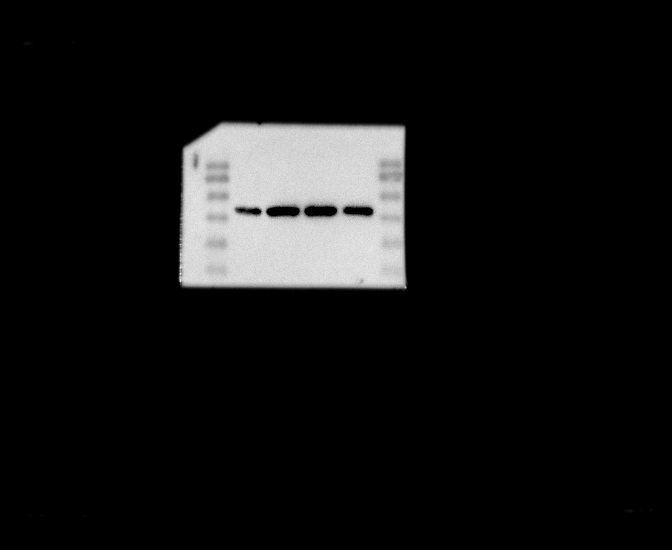

Supplement: Supplemental Information 1 [file peerj-11-15612-s001.zip › Raw data/Western Blot/6B/Slc2a1 (2).tif]

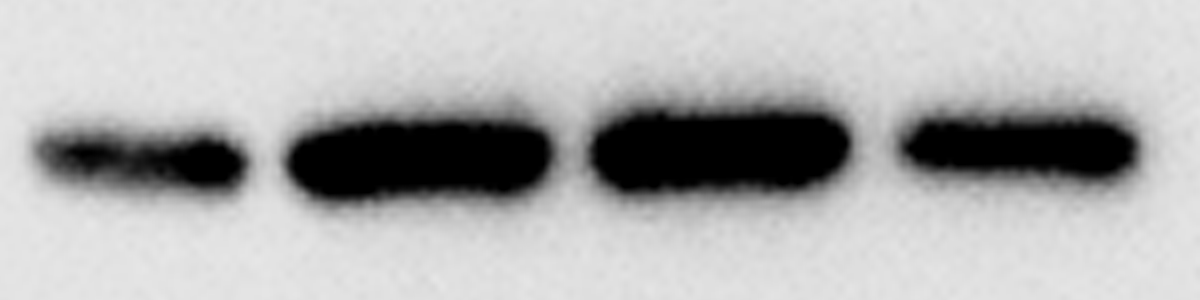

Supplement: Supplemental Information 1 [file peerj-11-15612-s001.zip › Raw data/Western Blot/6B/Slc2a1 (3).tif]

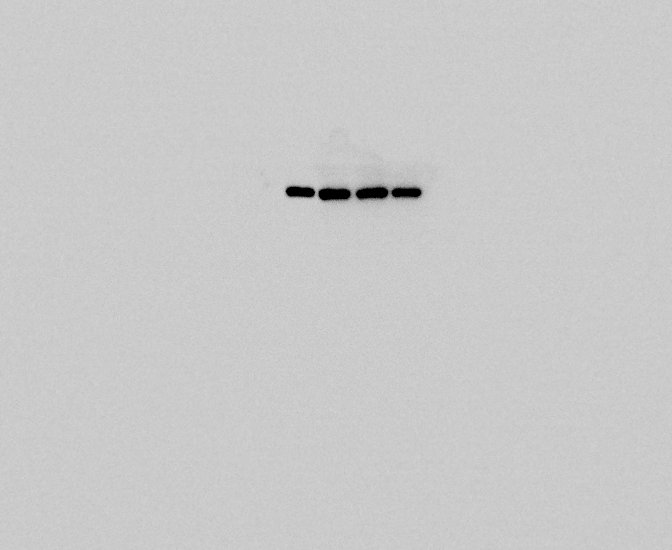

Supplement: Supplemental Information 1 [file peerj-11-15612-s001.zip › Raw data/Western Blot/6B/β-actin (1).tif]

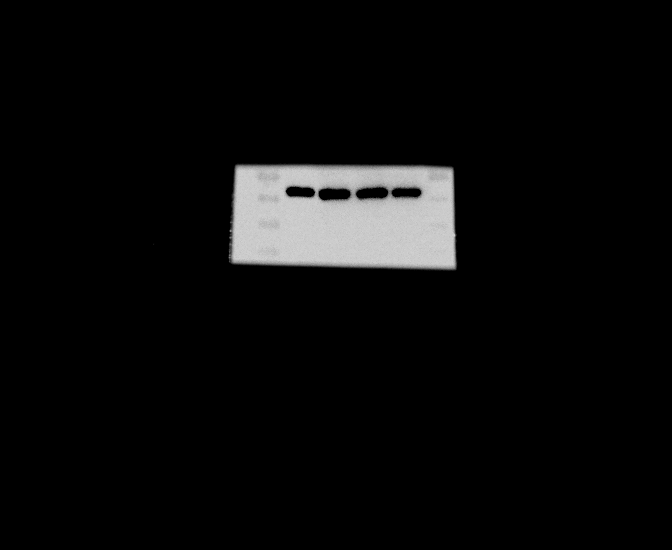

Supplement: Supplemental Information 1 [file peerj-11-15612-s001.zip › Raw data/Western Blot/6B/β-actin (2).tif]

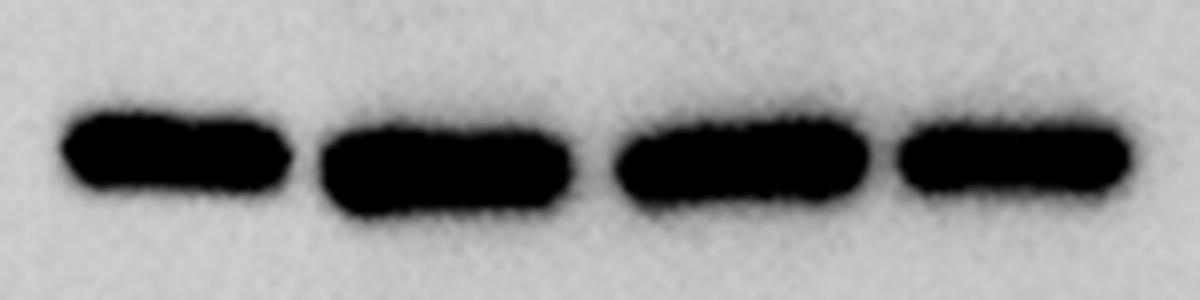

Supplement: Supplemental Information 1 [file peerj-11-15612-s001.zip › Raw data/Western Blot/6B/β-actin (3).tif]

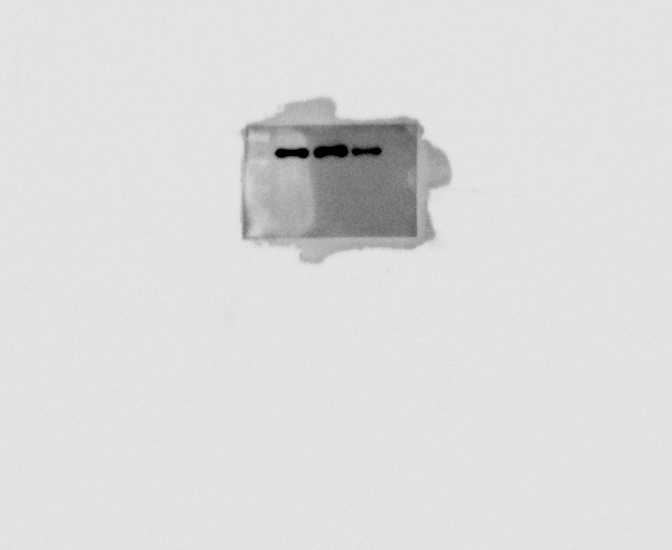

Supplement: Supplemental Information 1 [file peerj-11-15612-s001.zip › Raw data/Western Blot/7C/Slc2a1 (1).tif]

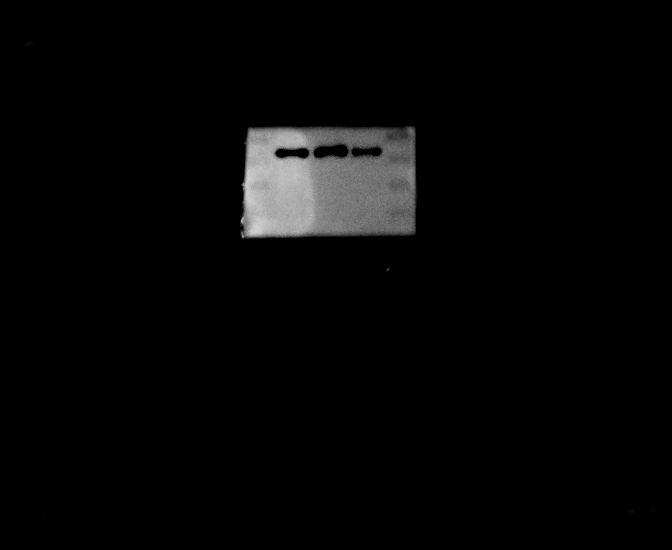

Supplement: Supplemental Information 1 [file peerj-11-15612-s001.zip › Raw data/Western Blot/7C/Slc2a1 (2).tif]

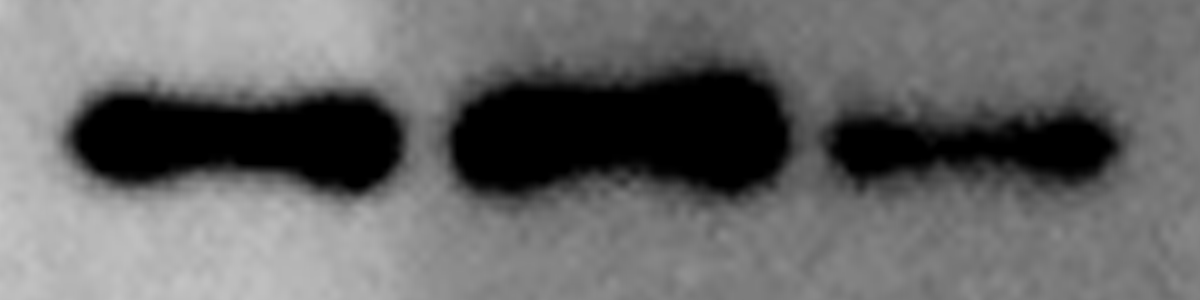

Supplement: Supplemental Information 1 [file peerj-11-15612-s001.zip › Raw data/Western Blot/7C/Slc2a1 (3).tif]

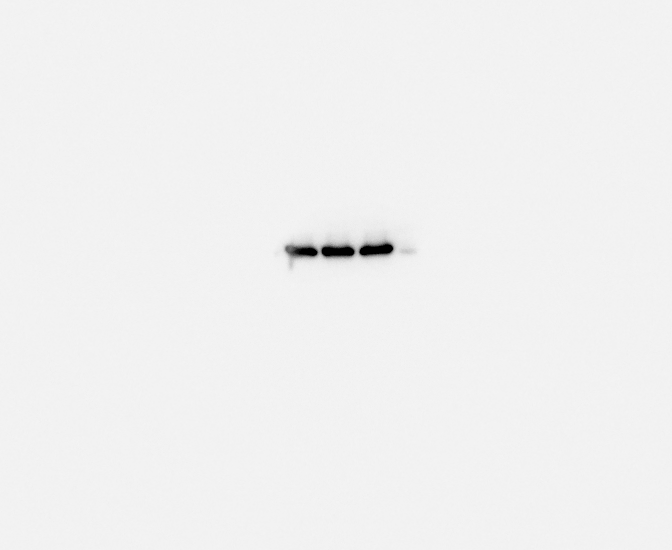

Supplement: Supplemental Information 1 [file peerj-11-15612-s001.zip › Raw data/Western Blot/7C/β-actin (1).tif]

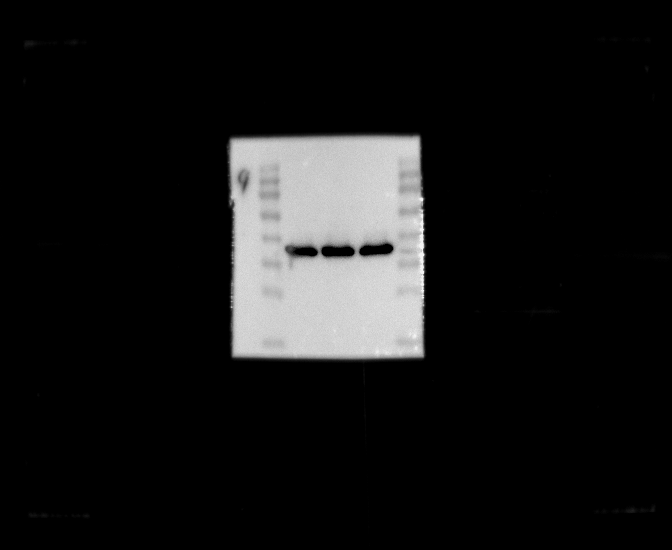

Supplement: Supplemental Information 1 [file peerj-11-15612-s001.zip › Raw data/Western Blot/7C/β-actin (2).tif]

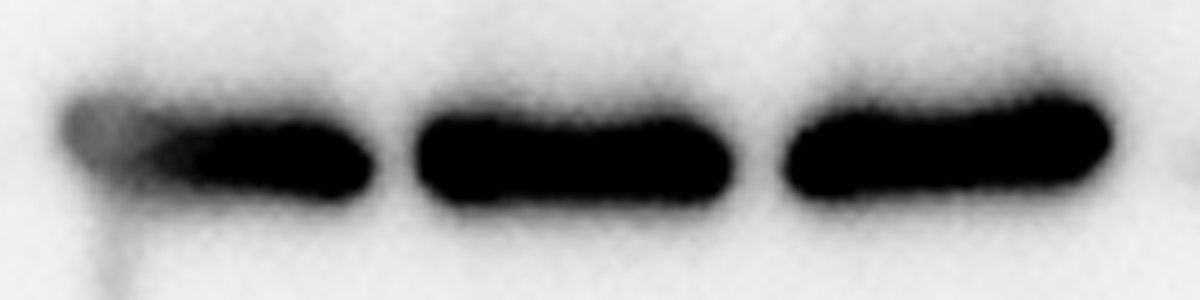

Supplement: Supplemental Information 1 [file peerj-11-15612-s001.zip › Raw data/Western Blot/7C/β-actin (3).tif]

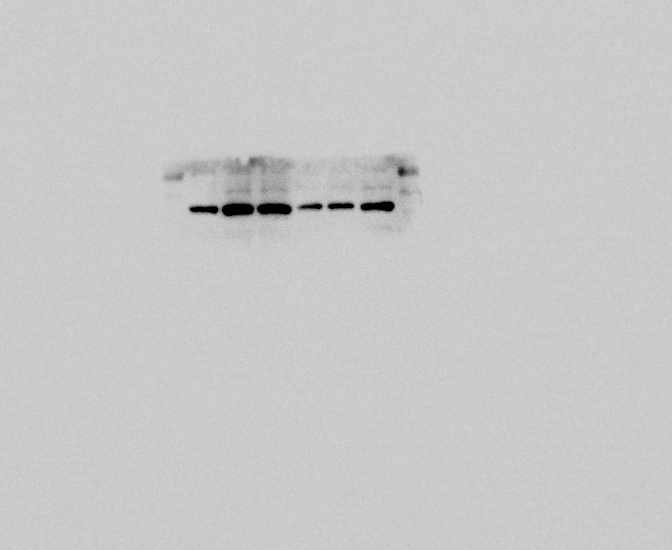

Supplement: Supplemental Information 1 [file peerj-11-15612-s001.zip › Raw data/Western Blot/8B/Slc2a1 (1).tif]

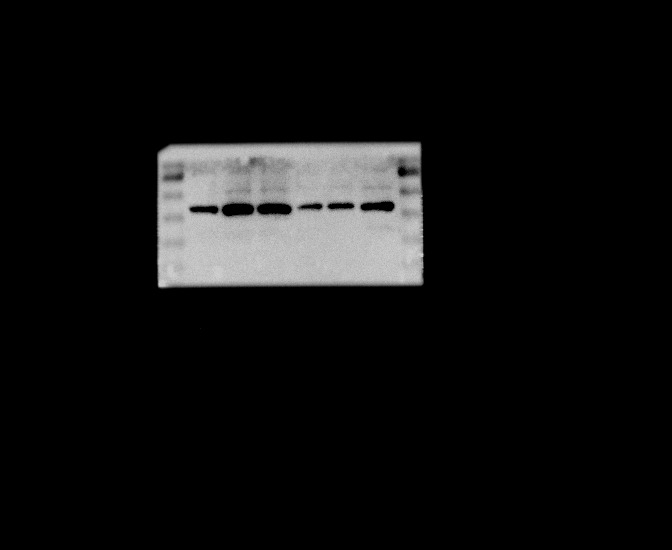

Supplement: Supplemental Information 1 [file peerj-11-15612-s001.zip › Raw data/Western Blot/8B/Slc2a1 (2).tif]

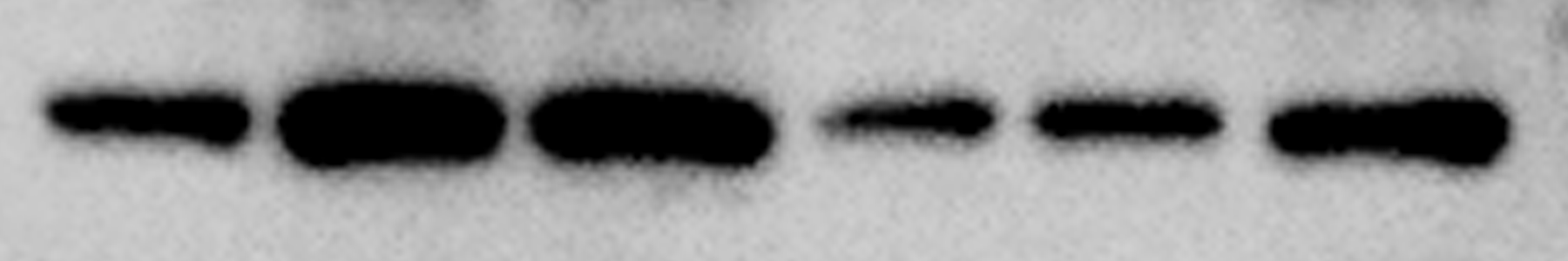

Supplement: Supplemental Information 1 [file peerj-11-15612-s001.zip › Raw data/Western Blot/8B/Slc2a1 (3).tif]

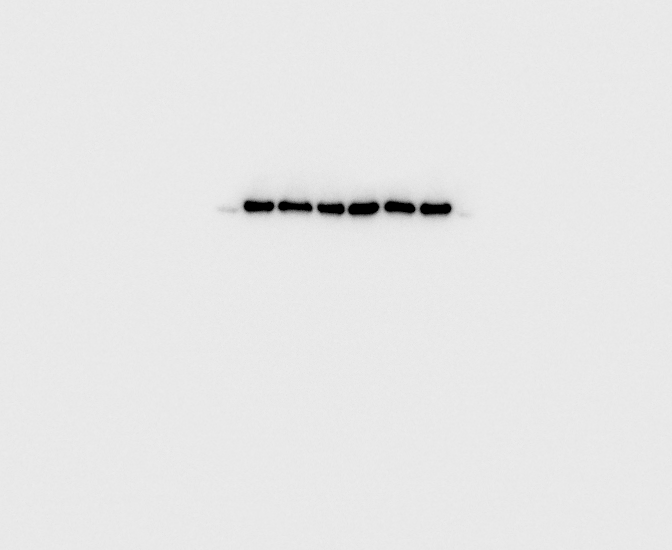

Supplement: Supplemental Information 1 [file peerj-11-15612-s001.zip › Raw data/Western Blot/8B/β-actin (1).tif]

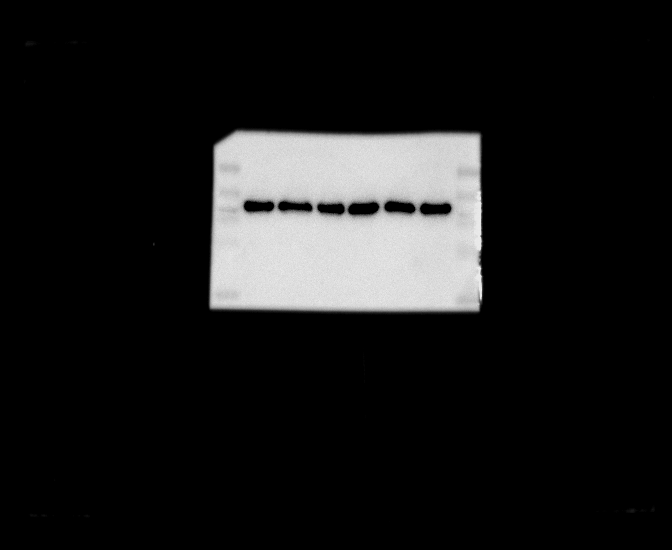

Supplement: Supplemental Information 1 [file peerj-11-15612-s001.zip › Raw data/Western Blot/8B/β-actin (2).tif]

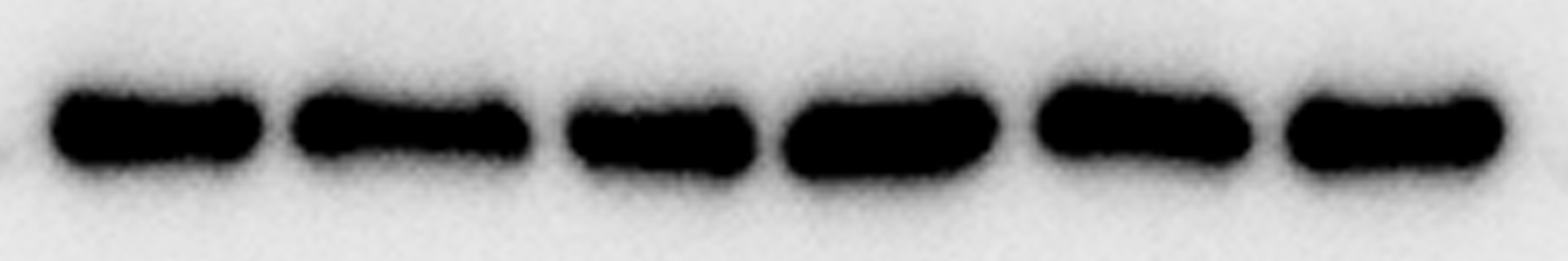

Supplement: Supplemental Information 1 [file peerj-11-15612-s001.zip › Raw data/Western Blot/8B/β-actin (3).tif]
